# High Phosphate and Low Protein Mediate Arterial and Cutaneous Vascular Calcification in CKD Mice

**DOI:** 10.1681/ASN.0000000875

**Published:** 2025-09-17

**Authors:** Ying Jin, Fei Cao, Yangzhouyun Xie, Sean Davis, Grace Dong, Sagar U. Nigwekar, James E. Hansen, Raul J. Guzman, Yujun Cai

**Affiliations:** 1 Division of Vascular Surgery and Endovascular Therapy, Department of Surgery, Yale School of Medicine, New Haven, Connecticut; 2 Department of Therapeutic Radiology, Yale School of Medicine, New Haven, Connecticut; 3 Division of Nephrology, Massachusetts General Hospital, Harvard Medical School, Boston, Massachusetts

**Keywords:** CKD, vascular calcification

## Abstract

**Key Points:**

- A high-phosphate and low-protein combination diet induced medial artery calcification, cutaneous vascular calcification, and kidney fibrosis in CKD mice.
- p38 mitogen-activated protein kinase signaling was highly activated in CKD mice fed a high-phosphate and low-protein diet.
- Pharmacologic p38 mitogen-activated protein kinase inhibition reduced vascular calcification and kidney fibrosis, suggesting a novel treatment for patients with CKD.

**Background:**

Medial artery calcification and cutaneous arteriolar calcification are prevalent in patients with CKD and are strongly associated with higher morbidity and mortality. Current experimental CKD models, however, often fail to fully replicate the patterns of medial artery calcification and cutaneous arteriolar calcification, limiting our ability to elucidate their underlying molecular pathways. Developing a reliable experimental model for CKD-associated calcification and using it to identify therapeutic targets is essential for advancing treatment strategies for these vascular complications. In this study, we used a novel strategy that incorporated a high-phosphate and low-protein (HPi-Lp) diet to promote medial artery and cutaneous vascular calcification in CKD mice.

**Methods:**

Mice underwent five-sixths (5/6) nephrectomy and were then fed various diets. Vascular calcification was assessed using micro–computed tomography scans, Alizarin Red staining, Von Kossa staining, and calcium assays. Kidney impairment and fibrosis were also evaluated. RNA sequencing analysis was performed to identify key molecular pathways. The pharmacologic inhibitor SB203580 was used to determine the significance of p38 mitogen-activated protein kinase (MAPK) signaling *in vivo*.

**Results:**

The HPi-Lp diet markedly induced both medial artery and cutaneous vascular calcification in 5/6 nephrectomized mice while exacerbating kidney dysfunction and fibrosis. The p38 MAPK signaling was specifically highly activated. Pharmacologic inhibition of p38 MAPK signaling significantly reduced medial artery and cutaneous vascular calcification as well as associated kidney fibrosis.

**Conclusions:**

The 5/6 nephrectomy CKD mouse model combined with a HPi-Lp diet effectively replicated medial artery and cutaneous arteriolar calcification.

## Visual Abstract

**Figure:**
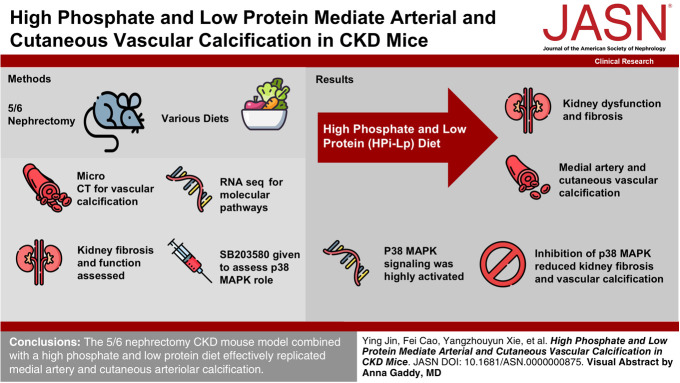

## Introduction

Vascular calcification is characterized by deposition of calcium hydroxyapatite crystals in the vessel wall. It is frequently encountered in individuals with CKD, atherosclerosis, and diabetes.^1–5^ There are four major types of vascular calcification, including atherosclerotic calcification, medial artery calcification, aortic valve calcification, and calciphylaxis. Based on the location of calcification within the vessel, it can be divided into two forms involving either the intima or the media.^6,7^ Intimal calcification commonly occurs in arteries affected by atherosclerosis, and it is believed to affect plaque stability.^7^ By contrast, medial calcification occurs within the elastic lamellae of the arterial media, resulting in vascular stiffening. It is particularly prevalent among individuals with kidney failure and chronic limb-threatening ischemia^8^ and is strongly associated with higher cardiovascular morbidity and mortality.^9^ Both forms of calcification are believed to share similar mechanisms and are tightly regulated by a series of endogenous pro-osteogenic and inhibitory factors.

Calciphylaxis, also called calcific uremic arteriolopathy, is a life-threatening condition associated with high mortality, particularly in individuals with kidney failure requiring dialysis.^10–14^ Patients with calciphylaxis often experience intense pain and have a poor prognosis, with an estimated median survival time of less than 1 year.^15^ It is now believed that calciphylaxis involves complex vascular and skin abnormalities, including cutaneous vascular calcification, intimal hyperplasia, fibrosis, inflammation, and tissue necrosis with skin ulceration.^16,17^ Therefore, inhibiting cutaneous vascular calcification could be beneficial in preventing and/or treating calciphylaxis.

Currently, two uremic mouse models, adenine-based and five/six nephrectomy (5/6 Nx) with a high-phosphate (Pi) diet, are commonly used to study calcification in CKD conditions.^18–21^ The adenine-enriched diet is unpalatable to rodents, resulting in variable food intake and significant interanimal variability. By contrast, the 5/6 Nx surgery reduces this variability, thus leading to more consistent experimental outcomes. In addition, CKD induced by excessive dietary adenine is nonphysiologic process, compared with 5/6 Nx mimicked progressive CKD as seen in human patients.^22^ Since C57BL/6J background mice are notoriously resistant to vascular calcification, these models typically require over 3 months to develop calcification, but the extent and frequency of medial calcification remained unsatisfactory.^19,21^ Previously, Price *et al.* showed that reducing dietary protein content from 25% to 2.5% in a 0.75% adenine diet dramatically increased the frequency and extent of medial artery calcification in rats.^23^ This finding led us to investigate whether low-protein intake is a critical factor in the development of medial artery calcification, a concept that has not yet been explored in C57BL/6J background mice. Considering the lack of research on cutaneous vascular calcification and therapeutic calcification models, the development of new animal models is urgently needed.

In this study, we aimed to develop a reliable mouse model of CKD-associated arterial and cutaneous vascular calcification and to determine whether inhibition of p38 mitogen-activated protein kinase (MAPK) signaling can reduce vascular calcification in this model.

## Methods

### Animals

All animal care and procedures were performed in compliance with protocols approved by the Yale University Institutional Animal Care and Use Committee (2022-20281). C57BL/6J mice were purchased from the Jackson Laboratory. 5/6 Nx surgery was performed as described previously.^24^ In brief, 12-week-old male C57BL/6J mice first underwent 2/3 pole ligation on the left kidney. After 1 week, the right kidney was removed. The Sham mice underwent similar procedures except that kidney ligation and removal were not performed. Animals were subcutaneously injected with extended-release buprenorphine (3.25 mg/kg) before surgery to relieve pain. After surgery, mice were recovered for 2 weeks and then randomly divided into four groups. Each group was fed one of three different diets for 4 weeks. These diets were high-phosphate and low-protein (HPi-Lp) diet (TD.180077) containing 2% phosphorus and 2.5% protein, high-phosphate–high-protein (HPi-Hp) diet (TD.220202) containing 2% phosphorus and 18% protein, and low-phosphate–low-protein (LPi-Lp) diet (TD.230207) containing 0.3% phosphorus and 2.5% protein. To study the role of p38 MAPK signaling in medial artery and cutaneous vascular calcification, 12-week-old male C57BL/6J mice underwent a 5/6 Nx or Sham procedure, followed by a 2-week recovery. The animals were fed either a HPi-Lp diet or a low-phosphate–high-protein diet (TD.230584) contained 0.3% phosphorus and 18% protein for 4 weeks. Two days before feeding the diet, mice underwent daily intraperitoneal injection with 10 mg/kg SB203580 or vehicle that continued until the end of the experiments. Animals were monitored daily and weighed once a week. All diets were purchased from Inotiv Envigo, and their detailed compositions are listed in Supplemental Table 1. The number of the animals used in the experiments was determined based on our pilot studies and the power analysis (PASS software 2019, Kaysville, UT; two tails; *α* error probability: 0.05; power: 0.8). The Animal Research: Reporting of *In Vivo* Experiments reporting guidelines have been used in this study.^25^

### Statistical Analysis

Values are mean±SD or mean±SEM. Data were analyzed using one-way or two-way ANOVA adjusted with the Tukey *post hoc* test for multiple comparisons. *P* < 0.05 was significant. Statistical analyses will be performed using GraphPad Prism 9 software.

Other detailed methods involved in the article are listed in Supplemental Methods. Primers used in the article are listed in Supplemental Table 2.

## Results

### Medial Artery Calcification and Cutaneous Arteriolar Calcification Were Induced in the 5/6 Nx Mice Fed the HPi-Lp Diet

**Figure 1:**
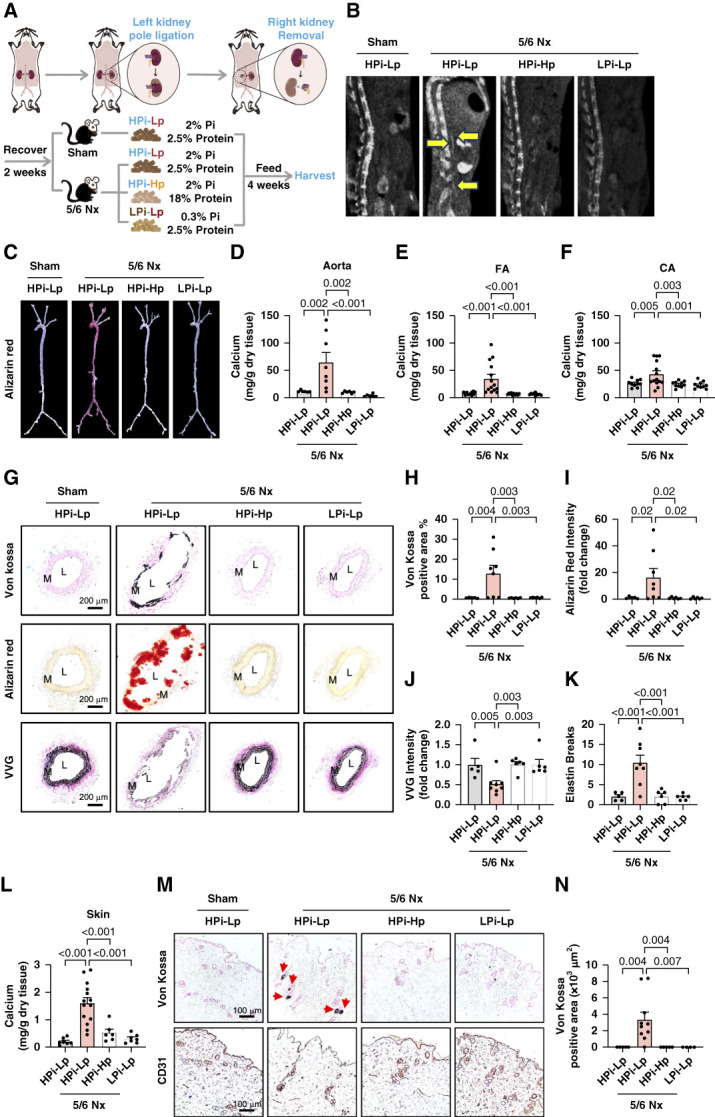
**Medial artery calcification and skin arteriolar calcification were induced in the 5/6 Nx mice fed the HPi-Lp diet.** (A) Schematic diagram of the animal procedures. Twelve-week-old male C57BL/6J mice underwent a two-step 5/6 Nx surgery to induce CKD. After 2 weeks of recovery, the 5/6 Nx mice were fed the different diets: HPi-Lp diet (2% phosphorus and 2.5% protein), HPi-Hp diet (2% phosphorus and 18% protein), and LPi-Lp diet (0.3% phosphorus and 2.5% protein) for 4 weeks. The Sham mice were fed a HPi-Lp diet without 5/6 Nx. (B) Micro-CT showed that calcification was increased in the thoracic and abdominal aortas of the 5/6 Nx-HPi-Lp mice compared with the other three groups. Yellow arrows indicated calcified vessels. (C) Representative images of Alizarin red staining showed that the HPi-Lp diet facilitated medial artery calcification in the 5/6 Nx mice. (D–F) Calcium content in the aorta, FA, and CA was robustly increased in the 5/6 Nx-HPi-Lp mice. Calcium content was examined using calcium assay. *n*=6–14. (G) Von Kossa, Alizarin red, and VVG staining showed that medial artery calcification was markedly increased in the abdominal aortas of the 5/6 Nx-HPi-Lp mice. (H–K) Quantitative data of Von Kossa, Alizarin red, and VVG staining. *n*=5–8. (L) Calcium assay showed that calcium content in the abdominal skin was largely increased in the 5/6 Nx-HPi-Lp mice. *n*=6–14. (M and N) Von Kossa staining and immunohistochemistry staining for the endothelial cell marker CD31 showed that calcium deposits were built up in the arterioles of the abdominal skin. Red arrows indicated calcified arterioles. *n*=4–10. Values are mean±SEM. Data were analyzed using one-way ANOVA adjusted with the Tukey *post hoc* test for multiple comparisons. *P* < 0.05 was significant. CA, carotid artery; CT, computed tomography; FA, femoral artery; HPi-Hp, high-phosphate–high-protein; HPi-Lp, high-phosphate–low-protein; L, lumen; LPi-Lp, low-phosphate–low-protein; M, media; 5/6 Nx, five/six nephrectomy; Pi, phosphate; VVG, Verhoeff–Van Gieson.

To examine the effect of Pi and dietary protein on medial calcification, 12-week-old C57BL/6J mice were subjected to 5/6 Nx to induce CKD. After 2 weeks, mice were fed a HPi-Lp diet, HPi-Hp diet, or LPi-Lp diet for 4 weeks (Figure 1A). Sham mice were fed the HPi-Lp diet without 5/6 Nx. During the experiment, we closely monitored the mice. No deaths occurred, and they remained active until harvesting. As shown in Supplemental Figure 1A, the 5/6 Nx-HPi-Lp mice experienced significantly greater weight loss compared with the other three groups. All mice fed a low-protein diet showed weight loss, regardless of whether they were CKD or Sham mice. However, the 5/6 Nx-HPi-Hp mice showed minimal weight changes during the feeding period, suggesting that the low-protein diet led to malnutrition in these mice. Malnutrition may contribute indirectly to vascular calcification by influencing systemic levels of calcification inhibitors (*e.g*., fetuin-A, matrix gla protein [MGP]) and bone-mineral metabolism.^26^ The combination of the 5/6 Nx and a HPi-Lp diet may create an ideal condition for maintaining activity while promoting malnutrition in the process of calcification formation. Using a micro–computed tomography scan, we observed that ectopic calcification was mainly located in thoracic and abdominal aortas of the 5/6 Nx-HPi-Lp mice (Figure 1B and Supplemental Figure 1B). We also calculated the bone volume of the tibia in these mice using 3D remodeling and found that the 5/6 Nx-HPi-Lp mice exhibited lower tibia bone volume compared with the other three groups (Supplemental Figure 1C). Next, we used Alizarin red staining to visualize calcium deposits in whole aortas. As shown in Figure 1C, strong calcification was observed in the entire aorta of the 5/6 Nx-HPi-Lp mice, but not the other three groups. Moreover, we assessed calcification in the aorta, femoral artery, carotid artery, kidney, heart, and lung. As shown in Figure 1, D–F, and Supplemental Figure 1, D–F, the 5/6 Nx-HPi-Lp mice had high calcification in all tested tissues compared with the other three groups. Similarly, Von Kossa and Alizarin red staining also showed strong calcification in the 5/6 Nx-HPi-Lp mice (Figure 1, G–I). Accordingly, we observed elastin lamellae degradation in these mice (Figure 1, G, J, and K). All these data suggested that 5/6 Nx with a HPi-Lp diet significantly induced arterial medial calcification.

Next, we examined whether cutaneous arteriolar calcification developed in the 5/6 Nx-HPi-Lp mice. As shown in Figure 1I, calcium content in the skin tissue was significantly increased in the 5/6 Nx-HPi-Lp mice compared with the other three groups. Von Kossa staining demonstrated calcified nodule formation in the dermis of the 5/6 Nx-HPi-Lp mice (Figure 1, M and N, and Supplemental Figure 1G). Immunohistochemistry staining for endothelial cell marker CD31 and smooth muscle cell marker smooth muscle alpha actin (SM-α-actin) further clarified that calcification occurred in the skin arterioles of the 5/6 Nx-HPi-Lp mice but not in the other three groups (Figure 1M and Supplemental Figure 1H). These data suggested that cutaneous arteriolar calcification developed in CKD mice fed a HPi-Lp diet. Tissue factor plays a critical role in vascular thrombosis during calciphylaxis.^27^ We further observed that tissue factor was markedly increased in calcified cutaneous vessels of CKD mice fed a HPi-Lp diet (Supplemental Figure 2, D and E). These findings suggest that our model shares pathophysiologic features of cutaneous arteriolar calcification in calciphylaxis.

### Smooth Muscle Cell Osteogenic Transformation and Vascular Inflammation Were Induced in the 5/6 Nx Mice Fed the HPi-Lp Diet

**Figure 2:**
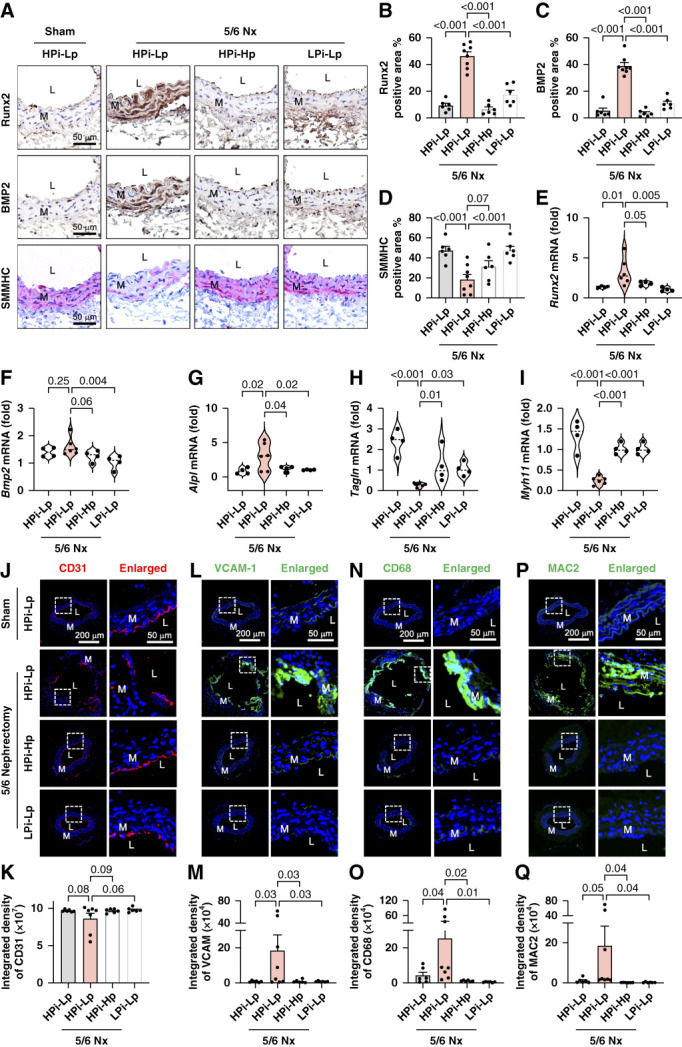
**Smooth muscle cell osteogenic transformation and vascular inflammation were induced in the 5/6 Nx mice fed the HPi-Lp diet.** Twelve-week-old male C57BL/6J mice underwent 5/6 Nx, and 2 weeks later were fed different diets for 4 weeks, as presented in Figure 1A. The abdominal aorta was dissected. (A) Immunohistochemistry staining showed that osteogenic transformation was induced in the abdominal aortas of the 5/6 Nx-HPi-Lp mice, showing increased osteogenic markers Runx2 and BMP2 and decreased smooth muscle cell contractile marker SMMHC. (B–D) Quantitative data of immunohistochemistry staining for Runx2, BMP2, and SMMHC. *n*=6 or 8. (E–I) qPCR results showed that the osteogenic genes *Runx2*, *Bmp2*, and *Alpl* were increased, and smooth muscle cell contractile genes *Tagln* and *Myh11* were decreased in the aortas of 5/6 Nx mice fed a HPi-Lp diet compared with the other groups. Gene expression levels were normalized to GAPDH and were presented as fold changes relative to the 5/6 Nx-LPi-Lp group. *n*=4 or 6. (J and K) Immunofluorescence staining for the endothelial cell marker CD31 showed a disturbed pattern of the endothelial layer in the aortas of the 5/6 Nx mice fed a HPi-Lp diet. *n*=6–8. (L and M) Immunofluorescence staining for VCAM-1 showed that vascular inflammation occurred in the 5/6 Nx-HPi-Lp mice. *n*=6–8. (N–Q) Immunofluorescence staining for CD68 and MAC2 showed macrophage accumulation in the aortas of the 5/6 Nx-HPi-Lp mice. *n*=6–8. Values are mean±SEM. Data were analyzed using one-way ANOVA adjusted with the Tukey *post hoc* test for multiple comparisons. *P* < 0.05 was significant. Nuclei were stained with DAPI. BMP2, bone morphogenetic protein 2; DAPI, 4′,6-diamidino-2-phenylindole; GAPDH, glyceraldehyde-3-phosphate dehydrogenase; MAC2, macrophage galactose–specific lectin 2; qPCR, quantitative PCR; Runx2, runt-related transcription factor 2; SMMHC, smooth muscle myosin heavy chain; VCAM-1, vascular cell adhesion molecule-1.

Since smooth muscle cell osteogenic transformation plays a pivotal role in vascular calcification,^28–32^ we next determined whether osteogenic transformation occurred in the 5/6 Nx CKD mice fed a HPi-Lp diet. As shown in Figure 2, A–D, the osteogenic markers, runt-related transcription factor 2 (Runx2) and bone morphogenetic protein 2 (BMP2), were increased, whereas contractile marker smooth muscle myosin heavy chain was decreased in the aortas of the 5/6 Nx-HPi-Lp mice compared with the other groups. Consistently, quantitative PCR data showed an increase in the osteogenic genes *Runx2*, *Bmp2*, and *Alpl* along with a decrease in the smooth muscle cell genes *Tagln* and *Myh11* in the aortas of these mice (Figure 2, E–I). These results suggested that smooth muscle cell osteogenic transformation occurs in the 5/6 Nx-HPi-Lp mice. In addition to smooth muscle cells, endothelial cells and macrophages are also involved in vascular calcification.^33,34^ Since CKD is an inflammatory disorder,^35^ we next examined changes in endothelial cells, macrophages, and inflammation in the 5/6 Nx-HPi-Lp mice. As shown in Figure 2, J and K, endothelial cell marker CD31 exhibited discontinuous and disturbed patterns in the abdominal aortas of the 5/6 Nx-HPi-Lp mice but remained intact in the other groups, suggesting that endothelial cells were affected during calcification. Next, we observed that vascular inflammation marker vascular cell adhesion molecule-1 was increased in the 5/6 Nx-HPi-Lp mice (Figure 2, I–M). Accordingly, macrophage markers CD68 and macrophage galactose-specific lectin 2 were increased in the calcified vessels (Figure 2, N–Q). These data suggest that endothelial cells and macrophages may play a role in the development of medial artery calcification in the 5/6 Nx-HPi-Lp mice. In addition, we examined the expression of Runx2 and BMP2 in the abdominal skins and found that Runx2 and BMP2 were significantly increased in the small vessels in the 5/6 Nx-HPi-Lp mice compared with the other groups (Supplemental Figure 2, A–C). These data suggest that osteogenic transformation occurs during cutaneous vascular calcification in the CKD mice fed a HPi-Lp diet.

### Glomerular Damage and Kidney Fibrosis Were Highly Induced in the 5/6 Nx Mice Fed the HPi-Lp Diet

**Figure 3:**
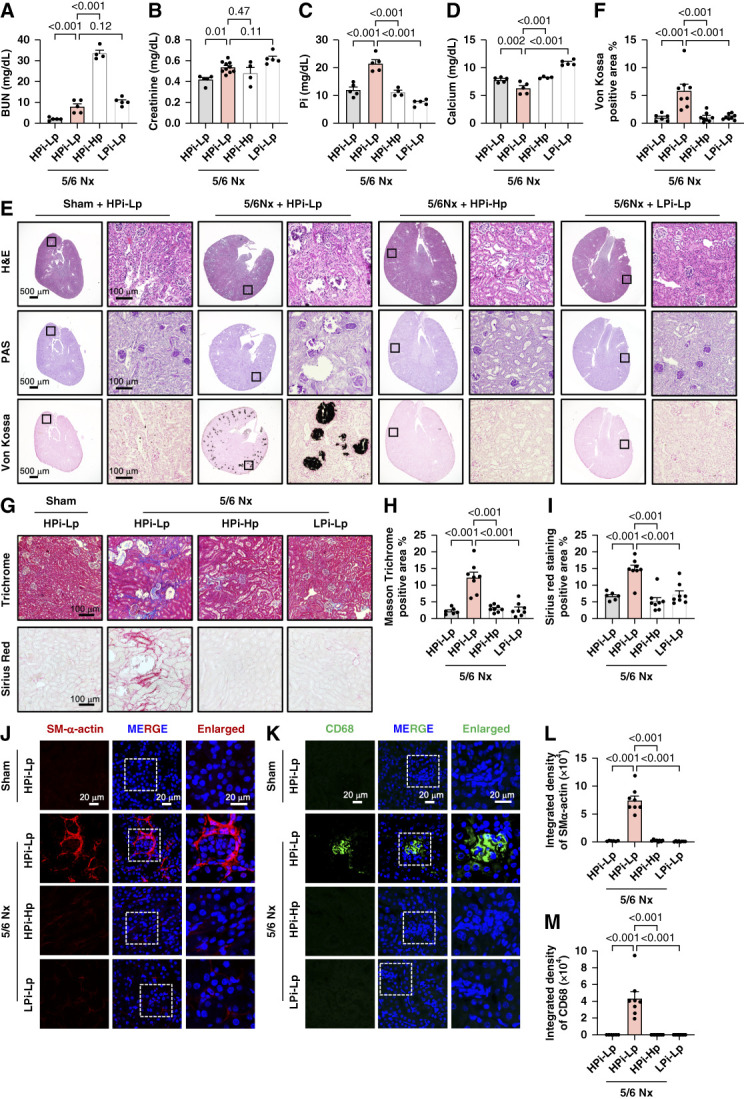
**Glomerular damage and kidney fibrosis were highly induced in the 5/6 Nx mice fed the HPi-Lp diet.** Twelve-week-old male C57BL/6J mice underwent 5/6 Nx, and 2 weeks later were fed the various diets for 4 weeks. Blood was collected, and the kidney was isolated. (A–D) Biochemical analysis of BUN, creatinine, Pi, and calcium in blood serum. *n*=4–10. (E) H&E, PAS, and Von Kossa staining showed that glomerulus damage and calcification were exacerbated in the 5/6 Nx-HPi-Lp mice compared with the other groups. (F) Quantitative data of Von Kossa staining. *n*=6 or 8. (G) Masson Trichrome and Sirius Red staining showed that the HPi-Lp diet increased kidney fibrosis in the kidney of the 5/6 Nx mice compared with the other diets. (H and I) Quantitative data of Masson Trichrome and Sirius Red staining. *n*=6 or 8. (J and L) Immunofluorescence staining showed that fibrosis marker SM-*α*-actin was induced in the kidney of the 5/6 Nx-HPi-Lp mice. *n*=6–8. (K and M) Immunofluorescence staining for macrophage marker CD68 showed that macrophages were involved in the kidney of the 5/6 Nx-HPi-Lp mice. *n*=6–8. Values are mean±SEM. Data were analyzed using one-way ANOVA adjusted with the Tukey *post hoc* test for multiple comparisons. *P* < 0.05 was significant. Nuclei were stained with DAPI. H&E, hematoxylin and eosin; PAS, periodic acid–Schiff; SM-α-actin, smooth muscle alpha actin.

Dietary protein and Pi are critical factors in patients with CKD.^36^ We first examined kidney function in the 5/6 Nx mice fed different diets. Compared with the HPi-Hp and LPi-Lp diet groups, mice fed the HPi-Lp diet exhibited significantly reduced serum albumin levels, whereas no difference was observed between Sham and 5/6 Nx mice fed the same HPi-Lp diet (Supplemental Figure 3A), suggesting the animals experienced hypoalbuminemia, a potential indicator of kidney dysfunction. As shown in Figure 3, A and B, and Supplemental Figure 3, B and D, all CKD mice fed different diets exhibited a significant increase in serum BUN, creatinine, parathyroid hormone (PTH), and cystatin C compared with Sham mice, indicating kidney injury in CKD mice. Notably, the 5/6 Nx-HPi-Hp group displayed the highest BUN levels among the four groups (Figure 3A), which aligned with previous findings that a high-protein diet elevated BUN levels.^37^ However, we did not observe significant differences in creatinine and cystatin C among various CKD groups (Figure 3B and Supplemental Figure 3D). Patients with CKD often experience hyperphosphatemia and hypocalcemia.^38^ In line with this, we observed that serum Pi levels were elevated and calcium levels were decreased in the 5/6 Nx-HPi-Lp mice compared with the other groups (Figure 3, C and D). Consistently, the expression of Pi transporter Pit-1 was increased, while Pit-2 was decreased in the aortas of the 5/6 Nx mice fed the HPi-Lp diet compared with the other three groups (Supplemental Figure 3, G and H). In addition, no significant differences in serum magnesium levels were observed among the four groups (Supplemental Figure 3C). The expression levels of MGP and fetuin-A in aorta samples were decreased in CKD mice compared with the Sham group, suggesting impaired kidney function and systemic mineral imbalance (Supplemental Figure 3, E and F). However, dietary variations among CKD mice did not result in significant differences in MGP or fetuin-A expression (Supplemental Figure 3, E and F).

We next evaluated changes in kidney histologic structure between these groups. As shown in Figure 3, E and F, periodic acid–Schiff and Von Kossa staining revealed that the basement membrane disruption and mineralization in the glomerulus were increased in the 5/6 Nx-HPi-Lp mice compared with the other groups. In addition, Masson Trichrome and Sirius Red staining revealed that kidney fibrosis was markedly increased in the 5/6 Nx mice fed the HPi-Lp diet, but not in those fed the HPi-Hp diet, LPi-Lp diet, or in the Sham mice (Figure 3, G–I). These data suggest that the HPi-Lp diet could accelerate kidney fibrosis in CKD mice. SM-*α*-actin is a well-established biomarker in tissue fibrosis.^39,40^ We further observed that SM-*α*-actin was dramatically increased in the 5/6 Nx-HPi-Lp mice (Figure 3, J and L). In addition, immunofluorescence staining revealed that the macrophage marker CD68 was primarily increased in the glomerulus of the 5/6 Nx-HPi-Lp mice (Figure 3, K and M), suggesting that this diet could promote kidney inflammation in these mice.

### Calcification-Related Genes Were Upregulated in the 5/6 Nx Mice Fed the HPi-Lp Diet

**Figure 4:**
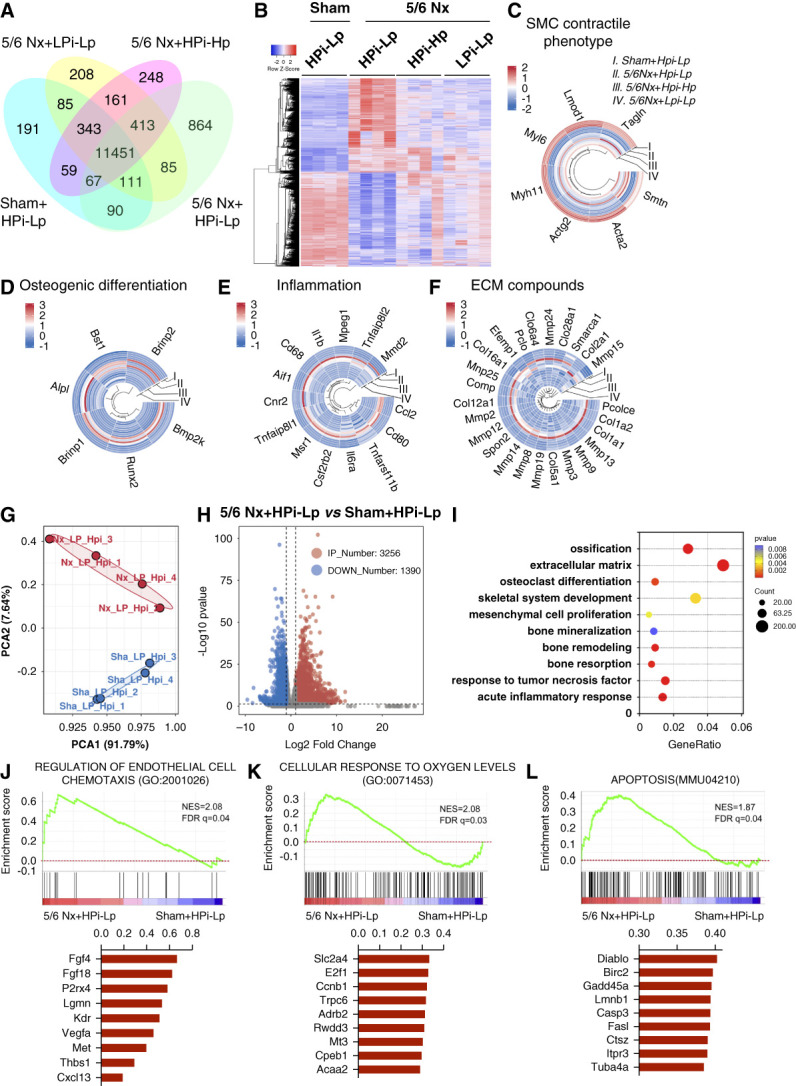
**Calcification-related genes were upregulated in the 5/6 Nx mice fed the HPi-Lp diet.** Twelve-week-old male C57BL/6J mice underwent 5/6 Nx, and 2 weeks later were fed the indicated diets for 4 weeks. Total RNA was isolated from the aortas for RNA-Seq. *n*=4 mice per group. (A) The Venn diagram of RNA-Seq data of the aortas from four groups. (B) Heat map of the top 1000 DEGs. (C–F). Circular heat maps showed that the HPi-Lp diet substantially downregulated genes associated with the smooth muscle cell contractile phenotype and upregulated genes related to osteogenic differentiation, inflammation, and the extracellular matrix in 5/6 Nx mice. (G) PCA on the gene expression value of the 5/6 Nx-HPi-Lp mice and the Sham animals. (H) Volcano plot showed 4646 DEGs between the 5/6 Nx-HPi-Lp and the Sham mice. (I) GO enrichment analysis showed multiple calcification-related pathways enriched between the 5/6 Nx-HPi-Lp and the Sham mice. (J–L) GSEA showed that endothelial cell chemotaxis, cell response to oxygen levels, and cell apoptosis were increased in the 5/6 Nx-HPi-Lp mice compared with the Sham animals. The top nine DEGs were listed. DEG, differently expressed gene; ECM, extracellular matrix; FDR, false discovery rate; GO, gene ontology; GSEA, gene set enrichment analysis; IP, intraperitoneal injection; NES, normalized enrichment score; PCA, principal component analysis; RNA-Seq, RNA sequencing; SMC, smooth muscle cell.

To explore the potential mechanisms responsible for medial artery calcification, we performed bulk RNA sequencing (RNA-Seq) analysis in the aortas collected from all four groups, as presented in Figure 1A. As shown in Figure 4A and Supplemental Figure 4, A and E, the Venn diagram showed that 11,451 genes were coexpressed among these four groups. The overlapping gene results of gene expression value cluster analysis showed that 3980 genes were uniquely upregulated in the 5/6 Nx-HPi-Lp mice, while 1467 genes were downregulated. A heat map of the top 1000 genes is shown in Figure 4B, and the detailed genes are listed in the Supplemental Table 3. Among these genes, the expression of genes related to smooth muscle contractile function was significantly downregulated in the 5/6 Nx-HPi-Lp mice compared with the other groups (Figure 4C). We also found that the genes associated with osteogenic differentiation, inflammation, and extracellular matrix compounds were uniquely and highly expressed in the 5/6 Nx-HPi-Lp mice (Figure 4, D–F). Next, we designated the 5/6 Nx-HPi-Lp mice as a calcification group and the other three groups as single-variable control groups, and pairwise analyses were conducted sequentially between the CKD calcification group and each control group. As shown in Figure 4G and Supplemental Figure 4, B and F, the principal component analysis on fragments per kilobase of transcript per million mapped reads showed that two groups dispersed and the samples within each group clustered closely, indicating that our data are reliable for further analysis. A total of 4646 differently expressed genes (DEGs; *P*_adj_ ≤ 0.05 and |log_2_FoldChange|≥1.0) were observed in the calcification group compared with the Sham group. Among these, 3256 genes were upregulated, and 1390 genes were downregulated (Figure 4H). Meanwhile, we observed that 2096 DEGs significantly altered in the calcification group compared with the 5/6 Nx-HPi-Hp mice, and a total of 2688 DEGs significantly altered in the calcification group compared with the 5/6 Nx-LPi-Lp mice (Supplemental Figure 4, C and G). Gene ontology analysis revealed that multiple calcification-related pathways, including ossification, bone mineralization, bone resorption, and inflammation, were enriched in the calcification mice compared with other three groups (Figure 4I and Supplemental Figure 4, D and H). Simultaneously, gene set enrichment analysis showed that the genes related to calcification, such as endothelial cell chemotaxis, cell response to oxygen levels, and cell apoptosis, were all upregulated in the calcification mice compared with the Sham mice (Figure 4, J–L). All these RNA-Seq data further confirmed that the HPi-Lp diet could increase arterial calcification, smooth muscle cell osteogenic transformation, and vascular inflammation in CKD mice.

### p38 MAPK Signaling Was Highly Activated in the 5/6 Nx Mice Fed the HPi-Lp Diet

**Figure 5:**
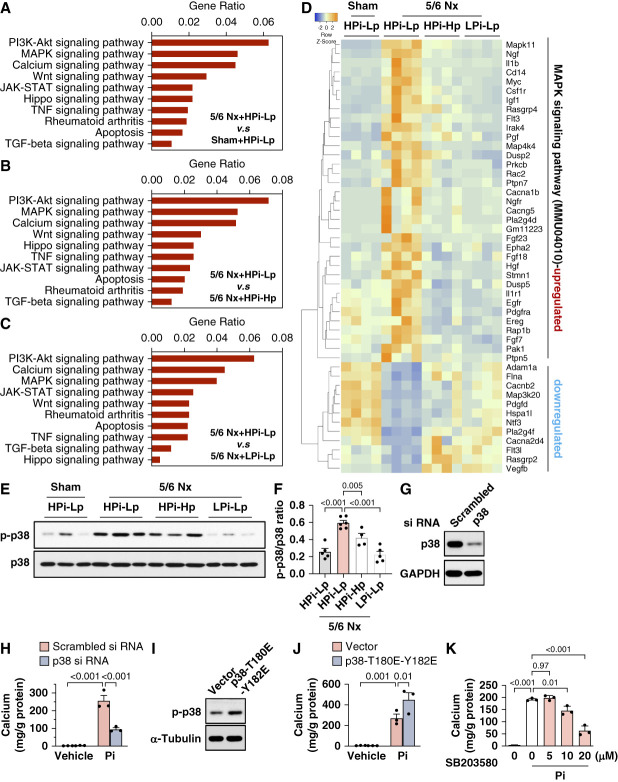
**p38 MAPK signaling was highly activated in the 5/6 Nx mice fed the HPi-Lp diet.** (A–C) KEGG analysis of calcification-related pathways in the aortas of the 5/6 Nx-HPi-Lp mice compared with the other three groups. (D) Heat map showed that MAPK signaling pathway was activated in the 5/6 Nx-HPi-Lp mice. (E and F) Western blotting showed that p-p38 MAPK was significantly higher in the aortas of the 5/6 Nx-HPi-Lp mice than in the other three groups. *n*=4–6. (G) Western blotting showed that p38*α* siRNA specifically decreased p38 in smooth muscle cells. Rat aortic smooth muscle cells were transfected with 50 nM p38*α* siRNA or scrambled siRNA for 3 days. GAPDH was used as an internal control. (H) The calcium assay data showed that knockdown of p38*α* inhibited Pi-mediated smooth muscle cell calcification. Rat aortic smooth muscle cells were transfected with 50 nM scrambled siRNA or p38*α* siRNA for 2 days and then cultured in a calcification medium containing 4.5 mM Pi for 6 days. *n*=3. (I and J) Calcium assay showed that activation of p38 significantly promoted Pi-mediated smooth muscle cells calcification. *n*=3. Rat aortic smooth muscle cells were transfected with 15 *μ*g pcDNA3.1-p38-T180E-Y182E or vector using electroporation, and p-p38 was confirmed using Western blotting. (K) p-p38 MAPK inhibitor SB203580 dose-dependently suppressed Pi-induced smooth muscle cell calcification. Rat aortic smooth muscle cells were pretreated with different concentrations of SB203580 and then treated with 4.5 mM Pi for 6 days. Calcium content was measured using a calcium assay. *n*=3. Values are mean±SEM for *in vivo* studies and mean±SD for *in vitro* experiments. Data were analyzed using one-way or two-way ANOVA adjusted with the Tukey *post hoc* test for multiple comparisons. *P* < 0.05 was significant. JAK-STAT, Janus kinase-signal transducer and activator of transcription; KEGG, Kyoto Encyclopedia of Genes and Genomes; MAPK, mitogen-activated protein kinase; PI3K, phosphoinositide 3-kinase; siRNA, small interfering RNA.

To define the mechanisms whereby the HPi-Lp diet promotes arterial calcification in CKD, we conducted a pairwise comparison of DEGs across designed groups using Kyoto Encyclopedia of Genes and Genomes pathway analysis (Figure 5, A–C). Among the top calcification-related pathways, we focused on the MAPK family signaling. As illustrated by the heat map, 35 of 47 genes classified in the MAPK signaling pathway (mmu04010) were upregulated in the calcified group compared with the other three groups (Figure 5D). Through gene set enrichment analysis, we further observed that MAPK family signaling was strongly activated in mice with calcification compared with those in the Sham group (Supplemental Figure 5A). Since p38 MAPK signaling plays a crucial role in the regulation of bone differentiation and bone formation,^41,42^ we examined the role of p38 MAPK signaling in medial artery and skin arteriolar calcification. p38 MAPK has four isoforms: p38*α*, p38*β*, p38*γ*, and p38*δ*. We found that p38*α* was the most abundant one among four p38 MAPK isoforms in smooth muscle cells and the aortas (Supplemental Figure 5, B and C). Next, we observed that p-p38 MAPK was dramatically increased in the calcified aortas of the 5/6 Nx-HPi-Lp mice compared with the other groups (Supplemental Figure 5, D and E). To identify the potential association between p-p38 MAPK expression and calcification levels in CKD mice, we plotted the percentage of p-p38 MAPK-positive area versus calcium content in the 5/6 Nx-HPi-Lp mice. As shown in Supplemental Figure 5F, there was a strong positive correlation between p-p38 expression and calcification extent in the aortas (R^2^=0.72, *P* = 0.0079). Western blotting data further confirmed that p-p38 MAPK was markedly increased in the 5/6 Nx-HPi-Lp mice compared with the other groups (Figure 5, E and F). To further determine the role of p38 in vascular calcification, we used p38*α* small interfering RNA. As shown in Figure 5, G and H, and Supplemental Figure 5, G–I, knockdown of p38*α* markedly attenuated Pi-induced smooth muscle cell calcification and osteogenic transformation. To determine whether p38 activation contributes to Pi-mediated smooth muscle cell calcification, we constructed a constitutively active p38 plasmid (p38-T180E-Y182E). We found that p38-T180E-Y182E promoted Pi-induced smooth muscle cell calcification, osteogenic transformation, and inflammation (Figure 5, I and J, and Supplemental Figure 5, J–N). SB203580 is a specific p-38 MAPK inhibitor recognized for its high selectivity toward p38*α* and p38*β*.^43,44^ As shown in Figure 5K, SB203580 treatment dose-dependently inhibited smooth muscle cell calcification. In addition, we also observed that p-p38 MAPK was increased in the small vessels of the abdominal skin and kidney section from the 5/6 Nx-HPi-Lp mice (Supplemental Figure 5, O–R).

### Inhibition of p38 MAPK Suppressed Medial Artery Calcification in the 5/6 Nx Mice Fed the HPi-Lp Diet

**Figure 6:**
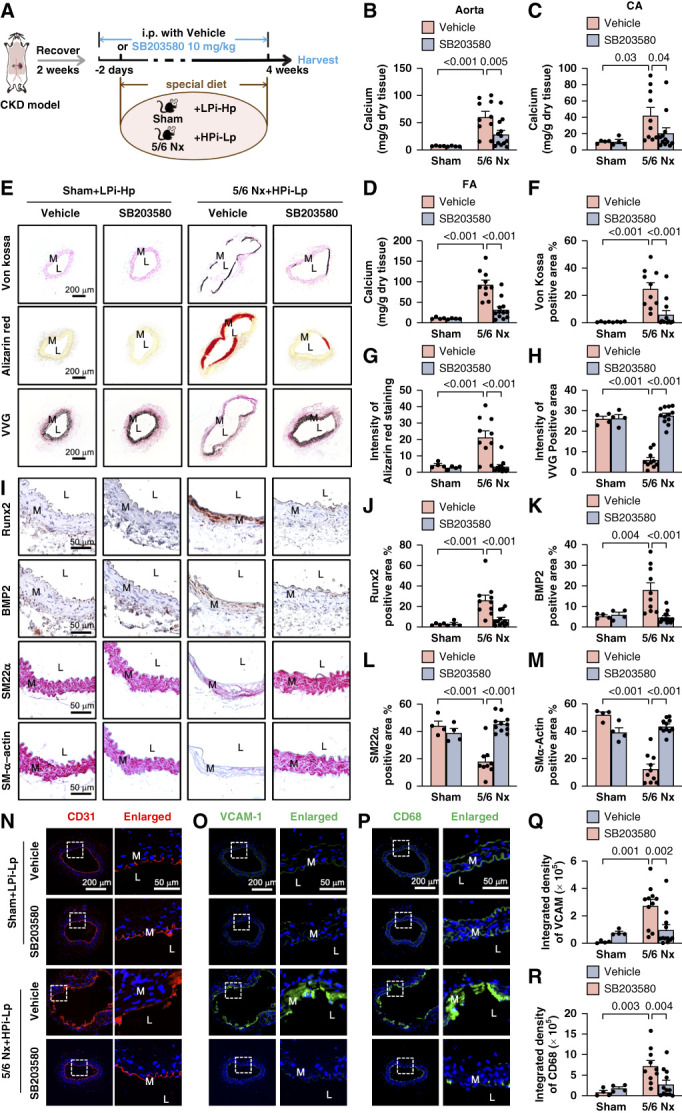
**Inactivation of p38 MAPK suppressed media artery calcification in the 5/6 Nx mice fed the HPi-Lp diet.** (A) Schematic diagram of SB203580 *in vivo* study. Twelve-week-old male C57BL/6J mice were subjected to 5/6 Nx or Sham surgery, and 2 weeks later fed a HPi-Lp diet or LPi-Hp diet for 4 weeks. p38 MAPK inhibitor SB203580 (10 mg/kg) or vehicle was daily IP injected 2 days before feeding the diet. The aortas were dissected. (B–D) Calcium assay showed that the administration of SB203580 significantly reduced calcium content in the aorta, FA, and CA. (E) Von Kossa, Alizarin red, and VVG staining showed that SB203580 administration suppressed medial artery calcification in the abdominal aortas of the 5/6 Nx-HPi-Lp mice. (F–H) Quantitative data of Von Kossa, Alizarin red, and VVG staining. (I) Immunohistochemistry staining showed that the administration of SB203580 inhibited osteogenic markers Runx2 and BMP2 and increased smooth muscle cell markers SM22*α* and SM-*α*-actin in the abdominal aortas of the 5/6 Nx-HPi-Lp mice. (J–M) Quantitative data of immunohistochemistry staining for Runx2, BMP2, SM22*α*, and SM-*α*-actin. (N) Immunofluorescence staining for CD31 showed that the administration of SB203580 blocked the disturbed endothelial layer in the abdominal aortas of the 5/6 Nx mice fed a HPi-Lp diet. (O and P) Immunofluorescence staining showed that the administration of SB203580 markedly suppressed VCAM-1 and CD68 in the abdominal aortas of the 5/6 Nx-HPi-Lp mice. Nuclei were stained with DAPI. (Q and R) Quantitative data of VCAM-1 and CD68. Values are mean±SEM. Sham group: *n*=4; 5/6 Nx groups: *n*=10 or 12 mice. Data were analyzed using two-way ANOVA adjusted with the Tukey *post hoc* test for multiple comparisons. *P* < 0.05 was significant. i.p., intraperitoneal; Lpi-Hp, low-phosphate–high-protein; SM22α, smooth muscle 22 alpha.

Next, we evaluated the effect of p38 MAPK inhibitor SB203580 on arterial calcification in the 5/6 Nx-HPi-Lp mice. The protocol is illustrated in Figure 6A. As shown in Supplemental Figure 6D, the administration of SB203580 slightly reduced weight loss compared with vehicle treatment in our CKD calcification model. During the experiment, we observed that two mice died in the vehicle CKD calcification group, whereas no animal died in the SB203580 CKD calcification group (Supplemental Figure 6E). As shown in Figure 6, B–D, and Supplemental Figure 6, A–C, calcium contents in all tested tissues were dramatically increased in the 5/6 Nx-HPi-Lp mice compared with the Sham mice. However, these effects were significantly blunted by the administration of SB203580. Von Kossa staining and Alizarin Red staining also showed that calcium deposits were markedly decreased by SB203580 administration compared with the vehicle in the 5/6 Nx-HPi-Lp mice (Figure 6, E–G). Accordingly, Verhoeff–Van Gieson staining showed that the administration of SB203580 blocked the degradation of elastic fibers (Figure 6, E and H). Furthermore, we observed that SB203580 markedly decreased smooth muscle cell osteogenic transformation as evidenced by decreased osteogenic markers Runx2 and BMP2 and increased smooth muscle cell markers smooth muscle 22 alpha (SM22α) and SM-*α*-actin in the 5/6 Nx-HPi-Lp mice (Figure 6, I–M). In addition, the administration of SB203580 inhibited the disruption of the endothelial layer and reduced CD68-positive and vascular cell adhesion molecule-1–positive staining, suggesting that inhibition of p38 MAPK could reduce inflammation in CKD calcification mice (Figure 6, N–R). Collectively, these data showed that the inactivation of p38 MAPK using SB203580 could significantly reduce medial artery calcification, osteogenic transformation, and inflammation in CKD mice.

### Inhibition of p38 MAPK Alleviated Cutaneous Arteriolar Calcification in the 5/6 Nx Mice Fed the HPi-Lp Diet

**Figure 7:**
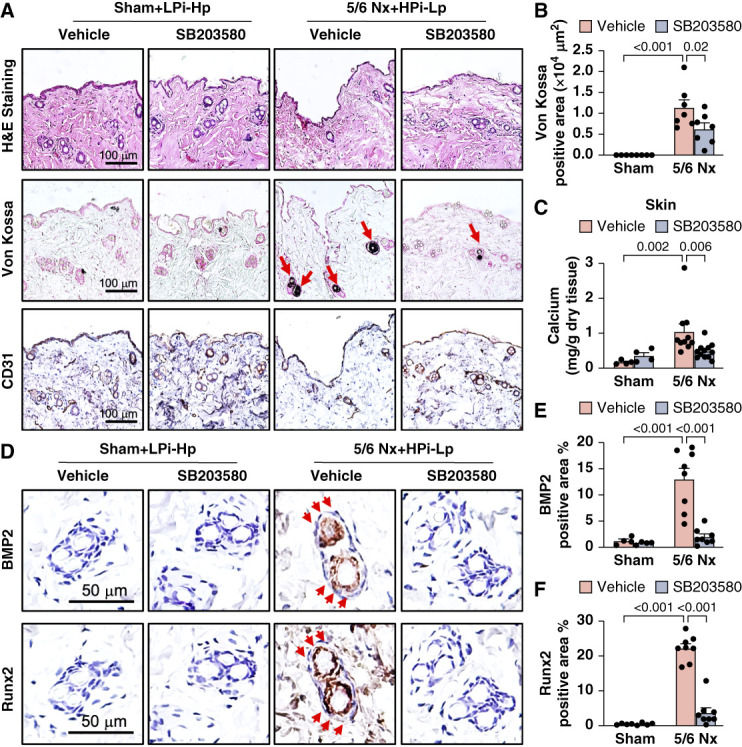
**Inhibition of p38 MAPK alleviated skin arteriolar calcification in the 5/6 Nx mice fed the HPi-Lp diet.** Twelve-week-old male C57BL/6J mice were subjected to 5/6 Nx or Sham surgery and then daily IP injected with 10 mg/kg SB203580 or vehicle, followed by a HPi-Lp diet or LPi-Hp diet feeding for 4 weeks, as presented in Figure 6A. The abdominal skins were collected. (A) H&E, Von Kossa staining, and immunohistochemistry for endothelial cell marker CD31 showed that calcium deposits in the arterioles of the abdominal skin of the 5/6 Nx-HPi-Lp mice. (B) Quantitative data of Von Kossa staining. *n*=4 or 7 mice. (C) Calcium assay showed that the administration of p38 MAPK inhibitor SB203580 significantly reduced calcium content in the 5/6 Nx-HPi-Lp mice compared with the vehicle. Sham: *n*=4; 5/6 Nx: *n*=10 or 12 mice. (D) The administration of SB203580 suppressed osteogenic transformation in the 5/6 Nx-HPi-Lp mice compared with the vehicle. The osteogenic markers Runx2 and BMP2 were examined using immunohistochemistry staining. (E and F) Quantitative data of immunohistochemistry staining. *n*=4 or 8 mice. Values are mean±SEM. Data were analyzed using two-way ANOVA adjusted with the Tukey *post hoc* test for multiple comparisons. *P* < 0.05 was significant.

Next, we explored the role of p38/MAPK signaling in cutaneous arteriolar calcification. Von Kossa staining and CD31 immunohistochemistry staining revealed that calcium was deposited in the arterioles of the skin in the 5/6 Nx-HPi-Lp mice (Figure 7, A and B). These effects, however, were markedly blunted by the administration of SB203580. Calcium assay also showed that calcium content in the skin was increased in the 5/6 Nx-HPi-Lp mice but was significantly suppressed in the SB203580-treated mice (Figure 7C). In addition, the expression of osteogenic markers Runx2 and BMP2 was increased in the skin arterioles of the 5/6 Nx-HPi-Lp mice, and this increase was significantly reduced by the administration of SB203580 (Figure 7, D–F). These data suggest that targeting p38 MAPK could be a promising therapeutic strategy for cutaneous arteriolar calcification.

### Inhibition of p38 MAPK Reduced Glomerular Damage and Kidney Fibrosis in the 5/6 Nx Mice Fed the HPi-Lp Diet

**Figure 8:**
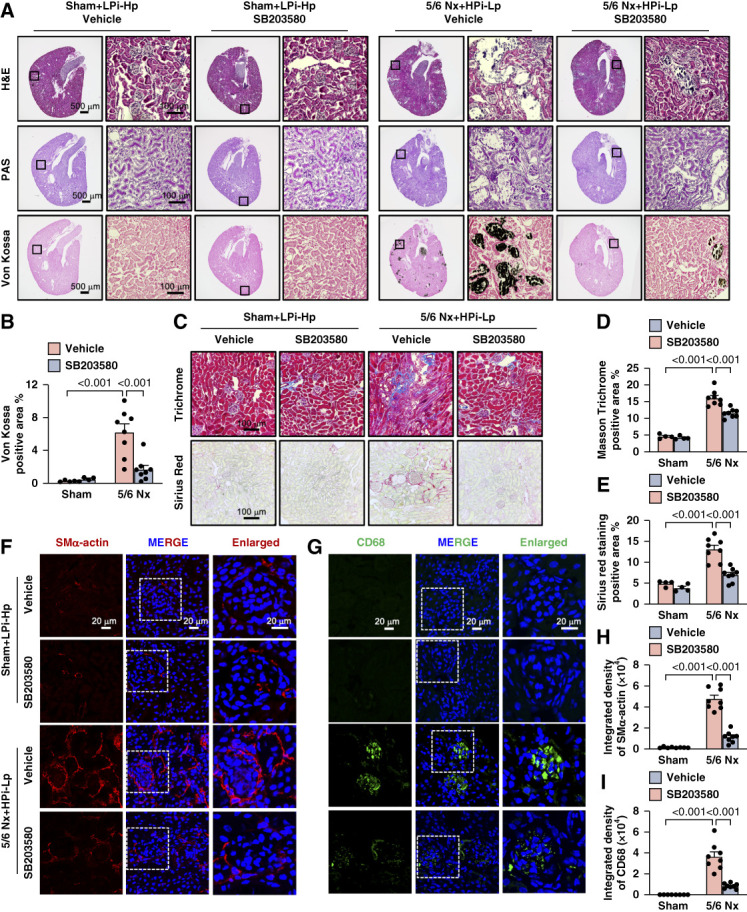
**Suppression of p38 MAPK reduced glomerular damage and kidney fibrosis in the 5/6 Nx mice fed the HPi-Lp diet.** Twelve-week-old male C57BL/6J mice were subjected to 5/6 Nx or Sham surgery and then IP injected with 10 mg/kg SB203580 or vehicle, followed by a HPi-Lp diet or LPi-Hp diet feeding for 4 weeks, as presented in Figure 6. The kidneys were isolated. (A) H&E, PAS, and Von Kossa staining showed that p38 MAPK inhibitor SB203580 suppressed glomerulus damage and calcification in the kidneys of the 5/6 Nx-HPi-Lp mice compared with the vehicle. (B) Quantitative data of Von Kossa staining. *n*=4 or 8 mice. (C–E) Masson's Trichrome and Sirius Red staining showed that the administration of SB203580 reduced kidney fibrosis in the kidneys of the 5/6 Nx-HPi-Lp mice. *n*=4 or 8 mice. (F and H) Immunofluorescence staining showed that SB203580 treatment significantly suppressed fibrosis marker SM-*α*-actin in the kidneys of the 5/6 Nx-HPi-Lp mice. *n*=4 or 8 mice. (G and I) Immunofluorescence staining for macrophage marker CD68 showed that SB203580 suppressed macrophage accumulation in the kidneys of the 5/6 Nx-HPi-Lp mice. *n*=4 or 8 mice. Values are mean±SEM. Data were analyzed using two-way ANOVA adjusted with the Tukey *post hoc* test for multiple comparisons. *P* < 0.05 was significant.

Because CKD mice fed a HPi-Lp exhibited extensive glomerular damage and kidney fibrosis, we next determined whether inhibition of p38 MAPK could suppress kidney dysfunction. Serum measurements in mice revealed that BUN levels were reduced in the SB203580 administration group compared with the vehicle group in CKD mice (Supplemental Figure 7A). However, no changes in PTH, creatinine, albumin, or Pi levels were observed in CKD mice after SB203580 administration compared with those receiving the vehicle (Supplemental Figure 7, B–E), suggesting that SB203580 did not mitigate hyperphosphatemia or kidney dysfunction in the 5/6 Nx-HPi-Lp model. Furthermore, the reduced serum calcium and fetuin-A levels in the 5/6 Nx-HPi-Lp group were restored by SB203580 administration, suggesting that SB203580 might protect against calcification by regulating calcium metabolism (Supplemental Figure 7, F and G). As shown in Figure 8, A and B, periodic acid–Schiff and Von Kossa staining revealed extensive glomerular impairments in the 5/6 Nx-HPi-Lp mice. By contrast, the administration of SB203580 alleviated these pathologic changes. Masson Trichrome and Sirius Red staining further showed that the fibrotic areas were significantly reduced by the administration of SB203580 in the 5/6 Nx-HPi-Lp mice (Figure 8, C–E). In addition, we observed that the administration of SB203580 markedly reduced myofibroblast marker SM-*α*-actin and macrophage marker CD68 in the 5/6 Nx-HPi-Lp mice (Figure 8, F–I). These data suggest that inhibition of p38 MAPK signaling could improve Pi-promoted kidney fibrosis.

## Discussion

The goals of this study were to investigate the roles of dietary Pi and protein in CKD-associated vascular calcification and to establish a reliable and effective mouse model for medial artery and cutaneous arteriolar calcification in C57BL/6J background. We found that the HPi-Lp diet could induce medial artery calcification, skin arteriolar calcium deposits, glomerular damage, and kidney fibrosis in the 5/6 Nx mice. Pharmacologic inhibition of p38 MAPK markedly suppressed medial artery and skin arteriolar calcification while improving kidney function. These findings suggest that HPi-Lp are critical for the development of medial artery and cutaneous arteriolar calcification in CKD mice, and targeting p38 MAPK signaling could be an effective therapeutic strategy for medial artery calcification and cutaneous vascular calcification in patients with CKD.

The molecular mechanisms underlying calciphylaxis remain largely unknown. Currently, no effective drug targets or therapies have been developed. One of the reasons is the lack of reliable mouse models that accurately reflect the clinical manifestations of human calciphylaxis. The first animal model associated with calciphylaxis was established in rats by Hans Selye in 1962.^14^ Calcification was induced by combining multiple factors, including sensitizing agents such as PTH, vitamin D3, calcium, and Pi, along with challenging factors such as trauma, iron, egg albumin, and glucocorticoids. In this model, cutaneous calcifications were present, but blood vessels were typically spared. The animals could shed the calcified skin and generate new skin, suggesting that this model does not fully recapitulate the human disease of calciphylaxis. Furthermore, no mouse model for calciphylaxis has been developed thus far. In this study, we observed mineralization and occlusion in the skin vessels of the 5/6 Nx-HPi-Lp mice after 4 weeks, suggesting that 5/6 Nx with HPi-Lp diet could be used as a mouse model for cutaneous vascular calcification. It is noteworthy that we did not observe cutaneous lesions or severe pain in this model within the time frame of this study. It has been reported that warfarin and vitamin D are associated with a higher risk of calciphylaxis in kidney failure patients, causing vascular calcification and skin necrosis.^45^ In the future, we may seek to alter our dietary regimen in an attempt to induce skin lesions to recapitulate human calciphylaxis.

Glomerular damage and interstitial fibrosis are key features of CKD. Both arterial calcification and nephrocalcinosis are common in patients with CKD, particularly those undergoing dialysis with kidney failure.^46^ In the 5/6 Nx-HPi-Lp mice, we observed increased calcium content in the kidney and robust calcium deposits in the glomerulus, suggesting that nephrocalcinosis could occur in this model. We speculate that endothelial cells within the glomerular capillaries may contribute to glomerular calcification. However, the underlying mechanisms require further investigation. Moreover, we observed that CD68-positive macrophages accumulated in the glomerulus, further suggesting that GN could be involved in our CKD calcification model. We observed significant kidney fibrosis in the 5/6 Nx-HPi-Lp mice but not in the 5/6 Nx mice fed either a HPi-Hp or a LPi-Lp diet. These data suggest that both HPi-Lp are essential for the robust induction of kidney fibrosis. In addition, we found that Pi levels in blood serum were significantly higher in the 5/6 Nx-HPi-Lp mice compared with the 5/6 Nx-HPi-Hp and 5/6 Nx-LPi-Lp mice. Hyperphosphatemia is common in CKD and can lead to vascular calcification and kidney fibrosis.^47^ Our data further suggest that 5/6 Nx with HPi-Lp might be used as a mouse model to study kidney fibrosis in CKD patients with hyperphosphatemia.

The p38 MAPK signaling pathway has been established to play a critical role in bone differentiation and bone formation.^41,48^ p38 MAPK is crucial for smooth muscle cell calcification *in vitro*.^49,50^ However, its effect on vascular calcification has barely been reported *in vivo*. Our study suggests that p38 MAPK signaling plays a critical role in medial artery calcification and cutaneous vascular calcification in the 5/6 Nx-HPi-Lp mice. We noticed that the 5/6 Nx mice fed a HPi-Lp exhibited more glomerular damage and kidney fibrosis than the 5/6 Nx mice fed a LPi-Lp diet, suggesting that high Pi levels could exacerbate kidney dysfunction, which is consistent with the concept that hyperphosphatemia contributes to the severity of CKD.^47,51^ It has been demonstrated that CKD, particularly kidney failure, can lead to hyperphosphatemia.^52,53^ Moreover, kidney damage may promote vascular calcification, as evidenced by the 5/6 Nx-HPi-Lp mice exhibiting significantly more severe calcification than the Sham mice on the same diet. This observation aligns with clinical findings.^54,55^ In addition, high Pi can mediate vascular calcification *in vitro* and *in vivo*, and increased vascular calcification further impairs kidney function. Therefore, hyperphosphatemia, vascular calcification, and kidney dysfunction likely interact together to exacerbate morbidity and mortality in patients with CKD. Notably, we observed that p38 MAPK inhibitor SB203580 administration was unable to reduce Pi levels in the 5/6 Nx-HPi-Lp mice while improving kidney fibrosis. This suggested that the antifibrotic effect of p38 inhibition might occur independently of Pi homeostasis, potentially through suppression of downstream inflammatory and fibrogenic pathways such as fetuin-A. Studies have shown that fetuin-A could reduce fibrosis and inflammation.^56,57^ Previous studies have also demonstrated that activating p38 MAPK signaling can enhance inflammatory responses that can be a major driving factor in kidney fibrosis.^58^ Inhibition of p38 MAPK could reduce kidney inflammation and interstitial fibrosis in unilateral ureteral obstruction model.^59,60^ Consistently, our data also showed that inflammation and kidney fibrosis were dramatically suppressed by p38 MAPK inhibition in 5/6 Nx-HPi-Lp mice.

The development of vascular calcification varies in complexity across different rodent species and strains.^61,62^ Compared with rats, mice generally have low susceptibility to vascular calcification. In mouse strains, the dolichos biflorus agglutinin/2J background is much less resistant to vascular calcification than the C57BL/6J background. Consequently, dolichos biflorus agglutinin/2J mice have been used in many calcification studies.^18,63–67^ However, the C57BL/6J strain is the most widely used, as most transgenic mice are generated on this background. C57BL/6J mice are resistant to vascular calcification, making it difficult to develop effective and reliable models. Inspired by Price's rat calcification model (0.92% phosphorus and 2.5% protein diet for 4 weeks), we adopted and optimized a diet of 2% phosphorus and 2.5% protein for use in 5/6 Nx mice, instead of the 0.75% adenine-induced CKD model. We found that the diet containing 2% phosphorus and 2.5% protein largely promoted medial artery calcification in CKD mice on C57BL/6J background, suggesting that dietary Pi and protein levels play a critical role in calcification development. Through comparison of the different diet compositions, we noted that although magnesium and calcium levels were not deliberately altered, the reduced Mg:Pi and Ca:Pi ratios may diminish protective effects and promote calcification—an aspect that warrants further investigation in future studies.

In summary, we demonstrated that a HPi-Lp diet promoted medial artery and cutaneous vascular calcification and exacerbated kidney dysfunction in CKD mice. We further showed that the inhibition of p38 MAPK signaling significantly alleviated these pathologic conditions. Our data suggest that the 5/6 Nx with HPi-Lp could be an effective and reliable mouse model for studying medial artery and cutaneous arteriolar calcification. In addition, our findings suggest that p38 MAPK plays a crucial role in both medial artery and cutaneous vascular calcification, and targeting p38 MAPK signaling could be a promising therapeutic strategy to mitigate these vascular disorders in patients with CKD.

## Supplementary Material

## Data Availability

Original data generated for the study are available in a public access repository. Data Type: Raw Data/Source Data. Repository Name: Gene Expression Omnibus. Linkable Citation: https://www.ncbi.nlm.nih.gov/geo. The dataset of transcriptome RNA-Seq has been deposited to Gene Expression Omnibus under accession code GSE288848. All data generated or analyzed in this study were included in the Supplemental Material and are available from the corresponding author on reasonable request.

## Declarative Statements

All animal experiments were conducted in accordance with the NIH Guide for the Care and Use of Laboratory Animals or an equivalent standard that meets or exceeds the ethical and welfare requirements outlined in the NIH Guide. All protocols were approved by the appropriate institutional animal care and use committee.

## Supplemental Material

Supplemental Methods

Supplemental Table 1. Detailed compositions of the diets.

Supplemental Table 2. The primers used for quantitative reverse transcription polymerase chain reaction.

Supplemental Table 3. Detailed list summarizing the top 1000 differentially expressed genes.

Supplemental Figure 1. Medial artery calcification was increased in the 5/6 Nx mice fed the HPi-Lp diet.

Supplemental Figure 2. The osteogenic transformation and tissue factor were increased in the skin of the 5/6 Nx mice fed the HPi-Lp diet.

Supplemental Figure 3. Kidney dysfunction was exacerbated in the 5/6 Nx mice fed the HPi-Lp diet.

Supplemental Figure 4. RNA-Seq analysis in the 5/6 Nx mice fed the HPi-Lp diet, HPi-Hp diet, or LPi-Lp diet.

Supplemental Figure 5. p38 MAPK signaling was activated in the 5/6 Nx mice fed the HPi-Lp diet.

Supplemental Figure 6. Inactivation of p38 mitogen-activated protein kinase reduced organ calcification, body weight loss, and animal mortality in the 5/6 Nx mice fed the HPi-Lp diet.

Supplemental Figure 7. Kidney function in the 5/6 Nx-HPi-Lp mice administrated with SB203580.
