# Supplementary material for: High Phosphate and Low Protein Mediate Arterial and Cutaneous Vascular Calcification in CKD Mice
Source: J Am Soc Nephrol. 2025 Sep 17;37(4):683–99. doi: 10.1681/ASN.0000000875 (PMC12448113; doi:10.1681/ASN.0000000875)
Supplement: Supplementary file 1 [file jasn-37-683-s001.pdf]

ASN American Society of Nephrology | JASN Journal of the American Society of Nephrology | KIDNEY360 Accessing Our World From Every Angle | CJASN Clinical Journal of the American Society of Nephrology

ASN Journal Disclosure Form

As per ASN journal policy, I have disclosed any financial relationships or commitments I have held in the past 36 months as included below. I have listed my Current Employer below to indicate there is a relationship requiring disclosure. If no relationship exists, my Current Employer is not listed.

Y. Cai reports the following:
Employer: Bayer;; and Ownership Interest: Bayer.

I understand that the information above will be published within the journal article, if accepted, and that failure to comply and/or to accurately and completely report the potential financial conflicts of interest could lead to the following: 1) Prior to publication, article rejection, or 2) Post-publication, sanctions ranging from, but not limited to, issuing a correction, reporting the inaccurate information to the authors' institution, banning authors from submitting work to ASN journals for varying lengths of time, and/or retraction of the published work.

Name: Yujun Cai
Manuscript ID: JASN-2025-000492
Manuscript Title: High phosphate and low protein mediate arterial and cutaneous vascular calcification in CKD mice
Date of Completion: July 10, 2025
Disclosure Updated Date: July 10, 2025

ASN Journal Disclosure Form

As per ASN journal policy, I have disclosed any financial relationships or commitments I have held in the past 36 months as included below. I have listed my Current Employer below to indicate there is a relationship requiring disclosure. If no relationship exists, my Current Employer is not listed.

F. Cao has nothing to disclose.

I understand that the information above will be published within the journal article, if accepted, and that failure to comply and/or to accurately and completely report the potential financial conflicts of interest could lead to the following: 1) Prior to publication, article rejection, or 2) Post-publication, sanctions ranging from, but not limited to, issuing a correction, reporting the inaccurate information to the authors' institution, banning authors from submitting work to ASN journals for varying lengths of time, and/or retraction of the published work.

Name: Fei Cao
Manuscript ID: JASN-2025-000492R1
Manuscript Title: High phosphate and low protein mediate arterial and cutaneous vascular calcification in CKD mice
Date of Completion: July 11, 2025
Disclosure Updated Date: July 11, 2025

ASN American Society of Nephrology
JASN Journal of the American Society of Nephrology
KIDNEY360 Accessing Our World From Every Angle
CJASN Clinical Journal of the American Society of Nephrology

ASN Journal Disclosure Form

As per ASN journal policy, I have disclosed any financial relationships or commitments I have held in the past 36 months as included below. I have listed my Current Employer below to indicate there is a relationship requiring disclosure. If no relationship exists, my Current Employer is not listed.

S. Davis reports the following:
Employer: Yale School of Medicine

I understand that the information above will be published within the journal article, if accepted, and that failure to comply and/or to accurately and completely report the potential financial conflicts of interest could lead to the following: 1) Prior to publication, article rejection, or 2) Post-publication, sanctions ranging from, but not limited to, issuing a correction, reporting the inaccurate information to the authors' institution, banning authors from submitting work to ASN journals for varying lengths of time, and/or retraction of the published work.

Name: Sean Davis
Manuscript ID: JASN-2025-000492R1
Manuscript Title: High phosphate and low protein mediate arterial and cutaneous vascular calcification in CKD mice
Date of Completion: July 10, 2025
Disclosure Updated Date: July 10, 2025

ASN
American Society of Nephrology
JASN
Journal of the American Society of Nephrology
KIDNEY360
Accessing Our World From Every Angle
CJASN
Clinical Journal of the American Society of Nephrology

ASN Journal Disclosure Form

As per ASN journal policy, I have disclosed any financial relationships or commitments I have held in the past 36 months as included below. I have listed my Current Employer below to indicate there is a relationship requiring disclosure. If no relationship exists, my Current Employer is not listed.

G. Dong reports the following:
Employer: Yale University

I understand that the information above will be published within the journal article, if accepted, and that failure to comply and/or to accurately and completely report the potential financial conflicts of interest could lead to the following: 1) Prior to publication, article rejection, or 2) Post-publication, sanctions ranging from, but not limited to, issuing a correction, reporting the inaccurate information to the authors' institution, banning authors from submitting work to ASN journals for varying lengths of time, and/or retraction of the published work.

Name: Grace Dong
Manuscript ID: JASN-2025-000492R1
Manuscript Title: High phosphate and low protein mediate arterial and cutaneous vascular calcification in CKD mice
Date of Completion: July 11, 2025
Disclosure Updated Date: July 11, 2025

ASN
American Society of Nephrology
JASN
Journal of the American Society of Nephrology
KIDNEY360
Accessing Our World From Every Angle
CJASN
Clinical Journal of the American Society of Nephrology

ASN Journal Disclosure Form

As per ASN journal policy, I have disclosed any financial relationships or commitments I have held in the past 36 months as included below. I have listed my Current Employer below to indicate there is a relationship requiring disclosure. If no relationship exists, my Current Employer is not listed.

R. Guzman reports the following:
Employer: Yale Medical School

I understand that the information above will be published within the journal article, if accepted, and that failure to comply and/or to accurately and completely report the potential financial conflicts of interest could lead to the following: 1) Prior to publication, article rejection, or 2) Post-publication, sanctions ranging from, but not limited to, issuing a correction, reporting the inaccurate information to the authors' institution, banning authors from submitting work to ASN journals for varying lengths of time, and/or retraction of the published work.

Name: Raul Guzman
Manuscript ID: JASN-2025-000492R2
Manuscript Title: High phosphate and low protein mediate arterial and cutaneous vascular calcification in CKD mice
Date of Completion: August 18, 2025
Disclosure Updated Date: August 18, 2025

ASN American Society of Nephrology
JASN Journal of the American Society of Nephrology
KIDNEY360 Accessing Our World From Every Angle
CJASN Clinical Journal of the American Society of Nephrology

ASN Journal Disclosure Form

As per ASN journal policy, I have disclosed any financial relationships or commitments I have held in the past 36 months as included below. I have listed my Current Employer below to indicate there is a relationship requiring disclosure. If no relationship exists, my Current Employer is not listed.

J. Hansen reports the following:
Employer: Yale School of Medicine; Consultancy: Patrys Ltd; Ownership Interest: Patrys Ltd; Research Funding: Patrys Ltd; and Patents or Royalties: Yale School of Medicine; Patrys Ltd.

I understand that the information above will be published within the journal article, if accepted, and that failure to comply and/or to accurately and completely report the potential financial conflicts of interest could lead to the following: 1) Prior to publication, article rejection, or 2) Post-publication, sanctions ranging from, but not limited to, issuing a correction, reporting the inaccurate information to the authors' institution, banning authors from submitting work to ASN journals for varying lengths of time, and/or retraction of the published work.

Name: James E Hansen
Manuscript ID: JASN-2025-000492R1
Manuscript Title: High phosphate and low protein mediate arterial and cutaneous vascular calcification in CKD mice
Date of Completion: July 11, 2025
Disclosure Updated Date: July 11, 2025

ASN American Society of Nephrology | JASN Journal of the American Society of Nephrology | KIDNEY360 Accessing Our World From Every Angle | CJASN Clinical Journal of the American Society of Nephrology

ASN Journal Disclosure Form

As per ASN journal policy, I have disclosed any financial relationships or commitments I have held in the past 36 months as included below. I have listed my Current Employer below to indicate there is a relationship requiring disclosure. If no relationship exists, my Current Employer is not listed.

Y. Jin has nothing to disclose.

I understand that the information above will be published within the journal article, if accepted, and that failure to comply and/or to accurately and completely report the potential financial conflicts of interest could lead to the following: 1) Prior to publication, article rejection, or 2) Post-publication, sanctions ranging from, but not limited to, issuing a correction, reporting the inaccurate information to the authors' institution, banning authors from submitting work to ASN journals for varying lengths of time, and/or retraction of the published work.

Name: Ying Jin
Manuscript ID: JASN-2025-000492R1
Manuscript Title: High phosphate and low protein mediate arterial and cutaneous vascular calcification in CKD mice
Date of Completion: July 11, 2025
Disclosure Updated Date: July 11, 2025

ASN American Society of Nephrology | JASN Journal of the American Society of Nephrology | KIDNEY360 Accessing Our World From Every Angle | CJASN Clinical Journal of the American Society of Nephrology

ASN Journal Disclosure Form

As per ASN journal policy, I have disclosed any financial relationships or commitments I have held in the past 36 months as included below. I have listed my Current Employer below to indicate there is a relationship requiring disclosure. If no relationship exists, my Current Employer is not listed.

S. Nigwekar reports the following:
Employer: Massachusetts General Hospital; Consultancy: Fresenius Medical Therapies Group; Epizon Pharma; Laboratoris Sanifit; Inozyme Pharma; CSL Vifor; Renibus Therapeutics; Renibus; Sanofi; Research Funding: Hope Pharma; Alnylam; Laboratoris Sanifit; Inozyme Pharma; CSL; Honoraria: Inozyme Pharma; Leadiant Pharma; Fresenius; Medpage; RubiconMD; Patents or Royalties: UpToDate ; Top 5 Questions to Ask Your Doctor (book); and Other Interests or Relationships: National Kidney Foundation (member, prior AKDH Journal AE, honoraria); American Society of Nephrology (member); NephSAP (honoraria); Mass General Brigham Innovation Fellowship (Sanofi).

I understand that the information above will be published within the journal article, if accepted, and that failure to comply and/or to accurately and completely report the potential financial conflicts of interest could lead to the following: 1) Prior to publication, article rejection, or 2) Post-publication, sanctions ranging from, but not limited to, issuing a correction, reporting the inaccurate information to the authors' institution, banning authors from submitting work to ASN journals for varying lengths of time, and/or retraction of the published work.

Name: Sagar U. Nigwekar
Manuscript ID: JASN-2025-000492R1
Manuscript Title: High phosphate and low protein mediate arterial and cutaneous vascular calcification in CKD mice
Date of Completion: July 12, 2025
Disclosure Updated Date: July 12, 2025

ASN American Society of Nephrology JASN Journal of the American Society of Nephrology KIDNEY360 Accessing Our World From Every Angle CJASN Clinical Journal of the American Society of Nephrology

ASN Journal Disclosure Form

As per ASN journal policy, I have disclosed any financial relationships or commitments I have held in the past 36 months as included below. I have listed my Current Employer below to indicate there is a relationship requiring disclosure. If no relationship exists, my Current Employer is not listed.

Y. Xie has nothing to disclose.

I understand that the information above will be published within the journal article, if accepted, and that failure to comply and/or to accurately and completely report the potential financial conflicts of interest could lead to the following: 1) Prior to publication, article rejection, or 2) Post-publication, sanctions ranging from, but not limited to, issuing a correction, reporting the inaccurate information to the authors' institution, banning authors from submitting work to ASN journals for varying lengths of time, and/or retraction of the published work.

Name: Yangzhouyun Xie
Manuscript ID: JASN-2025-000492R1
Manuscript Title: High phosphate and low protein mediate arterial and cutaneous vascular calcification in CKD mice
Date of Completion: July 11, 2025
Disclosure Updated Date: July 11, 2025
