# Supplementary material for: High Phosphate and Low Protein Mediate Arterial and Cutaneous Vascular Calcification in CKD Mice
Source: J Am Soc Nephrol. 2025 Sep 17;37(4):683–99. doi: 10.1681/ASN.0000000875 (PMC12448113; doi:10.1681/ASN.0000000875)
Supplement: Supplementary file 2 [file jasn-37-683-s002.pdf]

High phosphate and low protein mediate arterial and cutaneous vascular calcification in CKD mice

Ying Jin[1], Fei Cao[2], Yangzhouyun Xie[1], Sean Davis[1], Grace Dong[1], Sagar U. Nigwekar[3], James E. Hansen[2], Raul J. Guzman[1]* and Yujun Cai[1]*

[1]Division of Vascular Surgery and Endovascular Therapy, Department of Surgery, Yale School of Medicine, New Haven, CT 06510; [2]Department of Therapeutic Radiology, Yale School of Medicine, New Haven, CT 06510; [3]Division of Nephrology, Massachusetts General Hospital, Harvard Medical School, Boston, MA 02114.

Contents

Supplemental Methods
Supplemental Table 1. Detailed compositions of the diets
Supplemental Table 2. The primers used for qRT-PCR
Supplemental Table 3. Detailed list summarizing the top 1000 differentially expressed genes
Supplemental Figure 1. Medial artery calcification was increased in the 5/6 nephrectomy mice fed the HPi-Lp diet
Supplemental Figure 2. The osteogenic transformation and tissue factor were increased in the skin of the 5/6 nephrectomy mice fed the HPi-Lp diet
Supplemental Figure 3. Kidney dysfunction was exacerbated in the 5/6 nephrectomy mice fed the HPi-Lp diet
Supplemental Figure 4. RNA-seq analysis in the 5/6 nephrectomy mice fed the HPi-Lp diet, HPi-Hp diet, or LPi-Lp diet
Supplemental Figure 5. p38 MAPK signaling was activated in the 5/6 nephrectomy mice fed the HPi-Lp diet
Supplemental Figure 6. Inactivation of p38 MAPK reduced organ calcification, body weight loss, and animal mortality in the 5/6 nephrectomy mice fed the HPi-Lp diet
Supplemental Figure 7. Kidney function in the 5/6 nephrectomy-HPi-Lp mice administrated with SB203580

Supplemental Methods

Animals

To evaluate the effects of p38 MAPK inhibition on medial artery calcification and cutaneous vascular calcification, p38 MAPK pharmacological inhibitor SB203580 (AdooQ BioScience, A10824) was dissolved in 1.25 % (v/v) DMSO in saline. A 10 mg/kg dose of SB203580 is reported to be highly specific to p38 MAPK, with minimal impact on other targets;[1,2] therefore, we selected this dose for this study. For animal experiments, at least two different investigators were involved. One investigator performed the surgical procedure, while another investigator conducted the histology process. The number of biological replicates included in each experiment varied due to differences in sample availability and technical feasibility. Variations are noted in the figure legends.

Aortic smooth muscle cell culture and calcification

Rat aortic smooth muscle cells were isolated from the aortas of male Sprague-Dawley (SD) rats as previously described.[3] Smooth muscle cells were maintained in a DMEM medium containing 10% FBS and 1% pen/strep in a humidified, 37°C, 5% CO_2 incubator. To induce calcification, confluent smooth muscle cells were cultured in a calcification medium containing 4.5 mM Pi for 6 days. The calcification medium was replaced every 2 days.

siRNA knockdown

Rat p38α siRNA and scrambled siRNA were purchased from Millipore Sigma (CUGACAUAAUCCACAGGGA [dT][dT]). Rat aortic smooth muscle cells were transfected with 50 nM siRNA for 2 days using Lipofectamine RNAi/MAX reagent (Thermo Fisher Scientific, Waltham, MA). Knockdown efficiency was confirmed by western blotting using specific antibodies.

Construction and transfection of the constitutively active p38 plasmid

The p38 mutant p38-T180E-Y182E mimic constitutive activation was synthesized and subcloned into the pCDNA3.1 (Genscript). 15 μg pcDNA3.1-p38-T180E-Y182E or control vector was electroporated into the rat aortic smooth muscle cells. For electroporation, 1×10^6 cells were transfected with 15 μg plasmid at 300 V, 600 μF in a 4 mm cuvette using Gene Pulser Xcell Electroporation System (Bio-Rad).

Calcification assessment

Calcium levels were measured using the o-cresolphthalein complexone method as described

previously.[4,5] The dried aorta, carotid artery, femoral artery, heart, lung, and kidney, and rat aortic smooth muscle cells were incubated in 0.6 N HCl for 2 days, and a calcium assay was performed. Calcium levels in tissues were normalized with dried tissue weight and shown as mg/g dry aorta tissue, and calcium content in smooth muscle cells was normalized to total protein and shown as µg/mg protein. To visualize calcium deposits in the whole aorta, Alizarin red staining was performed. The intact aorta was carefully dissected, cleared of fat, and fixed in Zinc Formalin Fixative (Sigma-Aldrich, Z2902) for 2 days at 4°C. Next, the aorta was rinsed three times with PBS to remove the fixative solution and then stained with 0.0016% (wt/vol) Alizarin red S (Sigma-Aldrich, A5533) in 0.5% KOH for 1 day. The aorta was then destained in 0.05% KOH and photographed immediately. Additionally, Von Kossa staining and Alizarin red staining were used to examine calcification in the paraffin sections of the aorta and skin tissues.

Histological analysis

The abdominal aorta, skin of abdomen, and kidney dissected from mice were fixed with Zinc Formalin Fixative at 4°C overnight and then embedded with paraffin. Hematoxylin & eosin staining was performed for tissue morphology. Verhoeff-Van Gieson's (VVG) staining was utilized to evaluate elastin integrity. Masson trichrome and Sirius Red staining were used to examine kidney fibrosis.

RNA isolation and quantitative real-time PCR (qRT-PCR)

The entire aorta was collected from mice and immediately frozen in liquid nitrogen. Frozen aortas were ground into powder and then subjected to RNA extraction using RNeasy Mini Kit (Qiagen, Hilden, Germany). Total mRNA was reverse transcribed into cDNA with iScript cDNA Synthesis Kit (Bio-Rad, 170-8890). qRT-PCR was performed using PowerUp™ SYBR® Green Master Mix (ThermoFisher Scientific, A25742) in an Applied Biosystems 7500 Fast Real-Time PCR machine. The results were calculated using the $2^{-\Delta\Delta Ct}$ method and normalized with internal control GAPDH. The primers used for qRT-PCR were listed in **Supplemental Table 2**.

RNA-Seq analysis

RNA-Sequencing (RNA-Seq) was conducted by Novogene Corporation Inc (Sacramento, CA, USA). All raw reads were cleaned and mapped to the reference mouse genome (version mm10) using HISAT2 v2.0.5.[6] The counts of read numbers were calculated using featureCounts v1.5.0-p3[7] and further normalized to FPKM (Fragments Per Kilobase of transcript sequence per Millions base pairs sequenced). For the heat map, $\log_2$(FPKM+1)–transformed expression values for each

gene are presented. Detailed information, including gene names, gene IDs, and corresponding $\log_2$(FPKM+1) values, is provided in **Supplemental Table 3**. For differential expression analysis, the read count matrix was imported into the R package DESeq2 (version 1.20.0).[8] Genes with an absolute fold change of at least 2-fold (increase or decrease) and an adjusted P-value of ≤ 0.05 were considered significantly differentially expressed. Gene Ontology (GO) functional enrichment analysis and Kyoto Encyclopedia of Genes and Genomes (KEGG) enrichment analysis were conducted using the R package clusterProfiler (version 3.8.1).[9,10]

Immunofluorescence staining

The tissues were formalin-fixed and paraffin-embedded. The paraffin sections were deparaffinized and subjected to antigen retrieval with citrate buffer at 95°C for 30 min. Next, the sections were permeabilized with 0.2% Triton x-100/PBS for 15 min at room temperature and then blocked with Dako serum-free blocking solution (Dako, X090930) for 1 h. Immunofluorescence staining was performed by incubating a primary antibody overnight, washing with PBST (0.02% Tween 20/PBS), and incubating a secondary antibody Alexa Fluor 546 or 488 (ThermoFisher Scientific) for 1 h. Nuclei were stained with DAPI. The primary antibodies against VCAM (Santa Cruz, sc-13160), CD31 (R&D systems, AF3628), CD68 (Invitrogen, # MA5-13324), SM-α-Actin (Dako, M0851), SM22α (Invitrogen, # PA5-115925), Mac2 (Cedarlane, CL8942AP) were applied. The images were captured with a Zeiss AxioImager M1 confocal microscope. For subsequent analysis, the entire aortic vessel was imaged, while for kidney sections, more than five randomly selected fields were captured per mouse. Fluorescence images obtained by confocal microscopy were imported into ImageJ for quantitative analysis. Images were scaled according to the magnification, and a consistent threshold was applied to the desired positive channel to isolate specific staining. The integrated density of positive regions was measured and recorded for each image.

Immunohistochemical staining

The paraffin-embedded sections were deparaffinized, antigen retrieved, and endogenous peroxidase quenched by incubating with 3% hydrogen peroxide solution for 30 min. Next, the sections were blocked with Dako serum-free blocking solution (Dako, X090930) and incubated with a primary antibody, followed by a biotinylated secondary antibody and subsequent incubation with an ABC kit (Vector Laboratories, PK-4000). DAB substrate kit (Vector Laboratories, SK-4100) or Vector Red Substrate Kit were used to detect the signal. The sections were counterstained with hematoxylin (Vector Laboratories, H-3401-500). The primary antibody against Runx2 (MBL, D130-3), BMP2 (Abcam, ab14933), tissue factor (Abcam, ab318995), p-p38 (Invitrogen, #36-

8500), and CD31 (R&D systems, AF3628) were used. The images were captured with a bright field microscope. For aortic sections, the entire vessel was imaged for subsequent analysis. For kidney and skin sections, more than five random fields per mouse were captured. Images obtained by microscopy were imported into ImageJ for quantitative analysis. Images were scaled according to the magnification, and a consistent threshold was applied across all groups for each specific staining. The area of positive staining and the total tissue area were measured separately.

Western blot

The aorta was harvested from mice and snap-frozen in liquid nitrogen. The frozen aortas were then ground into powder and lysed in RIPA buffer supplemented with protease and phosphatase inhibitors (Invitrogen, 78443). Total protein content in the lysates was measured using the Pierce BCA Protein Assay Kit (Thermo Fisher Scientific, 23225). The protein lysates were separated on a 10% SDS-PAGE gel and transferred onto a PVDF membrane (Sigma Millipore, IPVH00010). The membrane was then blocked with 5% non-fat milk in PBST buffer, incubated overnight at 4°C with a primary antibody, followed by incubation with a horseradish peroxidase (HRP)-conjugated secondary antibody. Signal detection was performed using a Kodak film processor with ECL Western Blotting substrate (Thermo Fisher, 32106). The primary antibodies were anti-phospho-p38 (Cell Signaling Technology, #9211), anti-p38 (Cell Signaling Technology, #9212), anti-GAPDH (Millipore Sigma, MAB374) and anti-α-Tubulin (Millipore Sigma, T5168).

Kidney function measurements

Mice were anesthetized with 2.5% inhaled isoflurane via a precision vaporizer. Blood was collected through the right ventricle and left at room temperature for 1h to clot completely. Next, the samples were centrifuged at 10,000 rpm, 4 °C for 30 min, and the serum was obtained from the supernatant. The kidney function was detected by IDEXX Bioanalytics (Columbia, MO). Serum parathyroid hormone (PTH; Raybiotech, EIA-PTH-1) and Cystatin C (Invitrogen, EMCST3) levels were measured using an ELISA assay according to the manufacturer's protocol. Serum magnesium level was measured using colorimetric kit from Novus Biologicals (NBP3-25897).

Micro-computed tomography (micro-CT) scan and bone volume analysis

Micro-CT was used to detect bone volume. One day before harvesting, the mice underwent micro-CT imaging using the Small Animal Radiation Research Platform (Xstrahl Inc., GA, USA) to obtain the 3D reconstructed CT images. The bone volume of each mouse tibia was then calculated by the BoneJ plugin in imageJ as described previously.[11]

Supplemental Table 1. Compositions of the diets

Product Code	TD.230207	TD.230584	TD.180077	TD.220202
**Diet Composition**	2.5% Protein, 0.3% Pi, 1% Ca	18% Protein, 0.3% Pi, 1% Ca	2.5% Protein, 2% Pi, 1% Ca	18% Protein, 2% Pi, 1% Ca
**Formula (g/Kg)**				
**Casein**	28.0	206.89	28.0	206.89
**L-Cystine**	0.4	0.4	0.4	0.4
**Corn Starch**	402.073	259.67	370.16	207.42
**Maltodextrin**	130.0	130.0	130.0	130.0
**Sucrose**	273.267	244.08	251.58	244.08
**Soybean Oil**	50.0	46.0	50.0	46.0
**Cellulose**	50.0	50.0	50.0	50.0
**Mineral Mix, w/o Ca & P (98057)**	16.5	16.5	16.5	16.5
**Calcium Phosphate, monobasic, monohydrate**	11.4	6.3	57.0	53.0
**Calcium Carbonate**	20.5	22.4	2.5	3.75
**Sodium Phosphate, monobasic, monohydrate**	\	\	13.0	12.1
**Potassium Phosphate, monobasic**	\	\	13.0	12.1
**Vitamin Mix, ANI-93-VX (94047)**	15.0	15.0	15.0	15.0
**Choline Bitartrate**	2.75	2.75	2.75	2.75
**TBHQ, antioxidant**	0.01	0.01	0.01	0.01
**Nutrient Information**				
**Kcal/g**	3.6	3.6	3.4	3.4

Supplemental Table 2. The primers used for qRT-PCR

qPCR primers	Direction	Primer sequence
Mouse Runx2	Forward	5’-GCCACTTACCACAGAGCTATT-3’
	Reverse	5’-GAGGCGATCAGAGAACAAACT-3’
Mouse Bmp2	Forward	5’-CATTTAGAGGAGAACCCAGGTG-3’
	Reverse	5’-AGTCACTAGCAATGGCCTTATC-3’
Mouse Alpl	Forward	5’-GAACAGAACCGACGTGGAATA-3’
	Reverse	5’-TAGTGGGAATGCTTGTGTCTG-3’
Mouse Myh11	Forward	5’-AGAAGGAGCGAAACACAGAC-3’
	Reverse	5’-TGTCACATTAATCCCCACGAG-3’
Mouse Tagln	Forward	5’-CCAGACTGTTGACCTCTATGAAG-3’
	Reverse	5’-TCTTATGCTCCTGGGCTTTC-3’
Mouse MGP	Forward	5’-ACGAAAGCATGGAGTCCTATG-3’
	Reverse	5’-CTCTGTTGATCTCGTAGGCAG-3’
Mouse Fetuin-a	Forward	5’-TCAAAATGTGCCTCTCCCAG-3’
	Reverse	5’-GCATGAGATTTGCCTTGCAG-3’
Mouse Pit-1	Forward	5’-GTGTCCCTTCTCTTCCAGTTC-3’
	Reverse	5’-GTGTTGCCGCTTTTGTAGAG-3’
Mouse Pit-2	Forward	5’-CCTGCTCTTCCACTTCCTG-3’
	Reverse	5’-TCTTGTGTAACTCCGCCTTG-3’
Mouse GAPDH	Forward	5’-GTGGCAAAGTGGAGATTGTTG-3’
	Reverse	5’-CGTTGAATTTGCCGTGAGTG-3’
Rat MAPK14	Forward	5’-GACATAATCCACAGGGACCTAAA-3’
	Reverse	5’-TAGCCGGTCATTTCGTCATC-3’
Rat MAPK11	Forward	5’-CAGAAGGTGGCTGTGAAGAA-3’
	Reverse	5’-TGCTTCAGGTGTTTGAGTAGG-3’
Rat MAPK12	Forward	5’-GCATTAGCCCACCCATACTT-3’
	Reverse	5’-GTGCGGTCTACGTCATCAAA-3’
Rat MAPK13	Forward	5’-GACCGACCTGCAGAAGATAATG-3’
	Reverse	5’-CCAGCTGAGTGGATGTACTTTAG-3’
Rat GAPDH	Forward	5’-GATGCTGGTGCTGAGTATGT-3’
	Reverse	5’-GCGGAGATGATGACCCTTT-3’

Supplemental Table 3. Detailed list summarizing the top 1000 differentially expressed genes.

See attached Excel Supplemental Spreadsheet

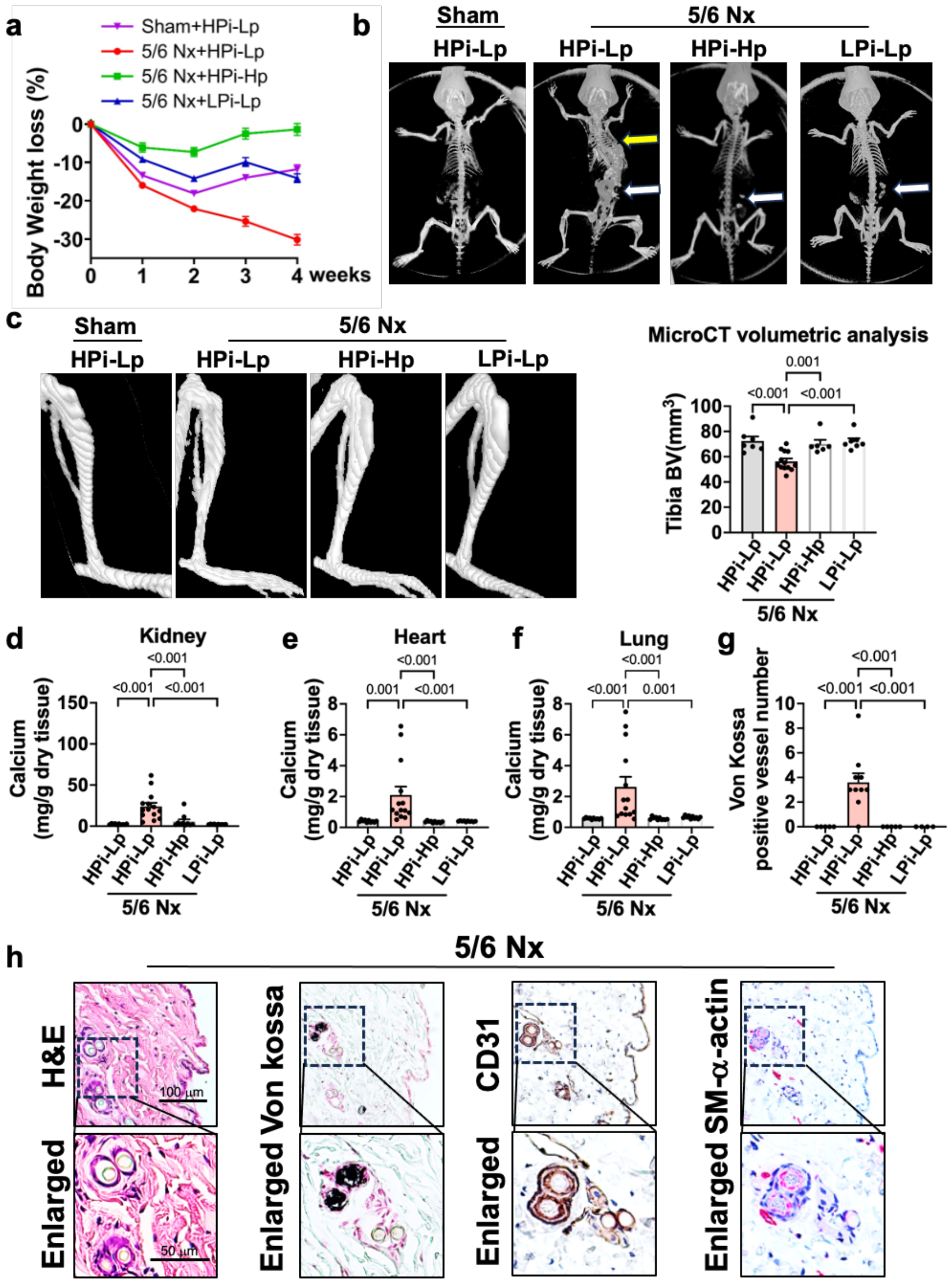

a
Sham+HPi-Lp
5/6 Nx+HPi-Lp
5/6 Nx+HPi-Hp
5/6 Nx+LPi-Lp
Body Weight loss (%)
weeks
b
Sham
5/6 Nx
HPi-Lp
HPi-Lp
HPi-Hp
LPi-Lp
c
Sham
5/6 Nx
HPi-Lp
HPi-Lp
HPi-Hp
LPi-Lp
MicroCT volumetric analysis
Tibia BV(mm³)
5/6 Nx
d
Kidney
Calcium (mg/g dry tissue)
e
Heart
f
Lung
g
Von Kossa positive vessel number
h
5/6 Nx
H&E
Von kossa
CD31
SM-α-actin
Enlarged
Enlarged Von kossa
Enlarged SM-α-actin
100 μm
50 μm

Supplemental Figure 1. Medial artery calcification was increased in the 5/6 nephrectomy mice fed the HPi-Lp diet. 12-week-old male C57BL/6J mice underwent 5/6 nephrectomy, and 2 weeks later, were fed the various diets for 4 weeks. (**a**) The effect of various diets on body weight in the 5/6 nephrectomy mice. (**b**) Micro-CT showed that calcification was induced in the whole-body soft tissues of the 5/6 nephrectomy-HPi-Lp mice compared with the other three groups. Yellow arrows indicated calcified areas, and white arrows indicated two ligated poles of the left kidney. (**c**) Micro-CT showed that tibia bone volume (BV) was reduced in the 5/6 nephrectomy-HPi-Lp mice compared with the other three groups. *n* = 6 to 13 mice. (**d-f**) Calcium assay showed that calcium contents in the kidney, heart, and lung were increased in the 5/6 nephrectomy-HPi-Lp mice. *n* = 10 or 14 mice. (**g**) Von Kossa staining showed that the number of calcifying arterioles in the abdominal skin was increased in the 5/6 nephrectomy-HPi-Lp mice compared with the other three groups. *n* = 4 to 10. (**h**) H&E staining, Von Kossa staining, and immunohistochemistry staining for the smooth muscle cell marker SM-α-actin and the endothelial cell marker CD31 showed that calcification occurred in arterioles of abdominal skin in the 5/6 nephrectomy-HPi-Lp mice. Values are mean $\pm$ SEM. Data were analyzed using one-way ANOVA adjusted with Tukey's post-hoc test for multiple comparisons. $P<0.05$ was significant. 5/6 Nx: 5/6 nephrectomy.

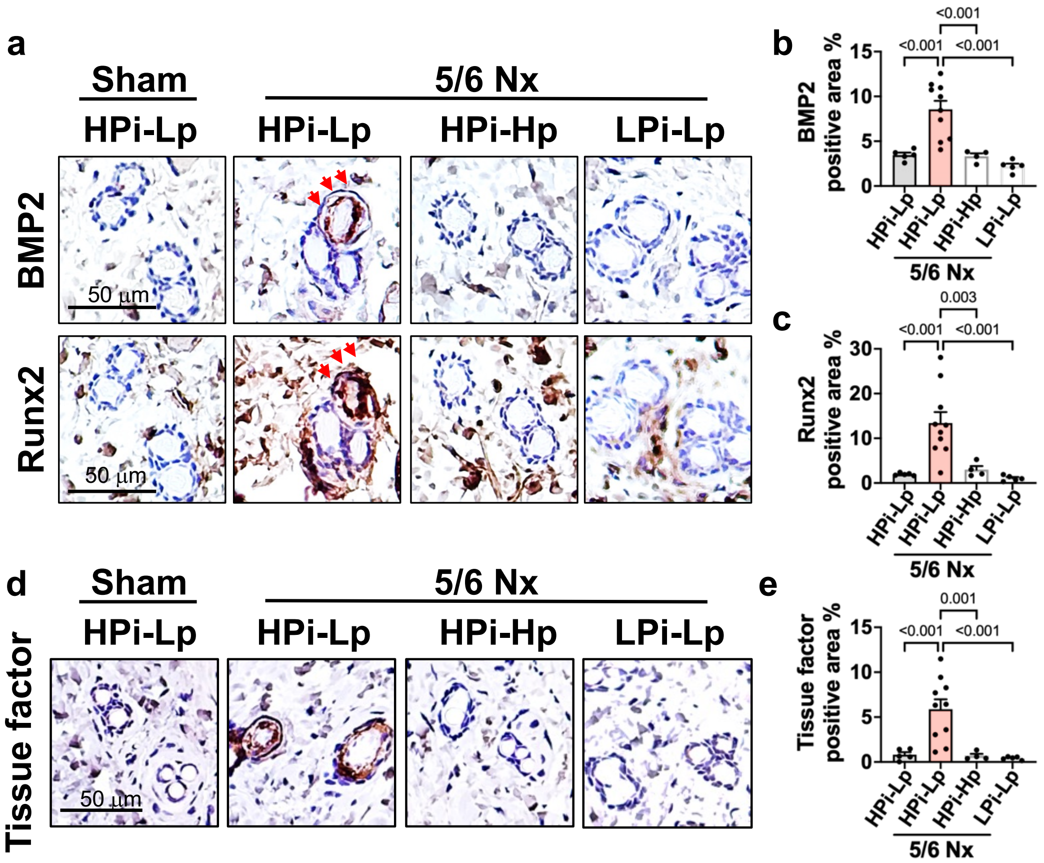

Supplemental Figure 2. The osteogenic transformation and tissue factor were increased in the skin of the 5/6 nephrectomy mice fed the HPi-Lp diet. 12-week-old male C57BL/6J mice underwent 5/6 nephrectomy, and 2 weeks later, were fed the various diets for 4 weeks. The abdominal skin tissues were collected. (**a**) Immunohistochemistry staining showed that the osteogenic markers BMP2 and Runx2 were increased in the arterioles of the abdominal skins from the 5/6 nephrectomy-HPi-Lp mice. (**b-c**) Quantitative data of immunohistochemistry staining for BMP2 and Runx2. *n* = 4 to 10. (**d**) Immunohistochemistry staining showed that tissue factor was increased in the arterioles of the abdominal skins from the 5/6 nephrectomy-HPi-Lp mice. (**e**) Quantitative data of immunohistochemistry staining for tissue factor. *n* = 4 to 10. Values are mean ± SEM. Data were analyzed using one-way ANOVA adjusted with Tukey's post-hoc test for multiple comparisons. *P*<0.05 was significant. 5/6 Nx: 5/6 nephrectomy.

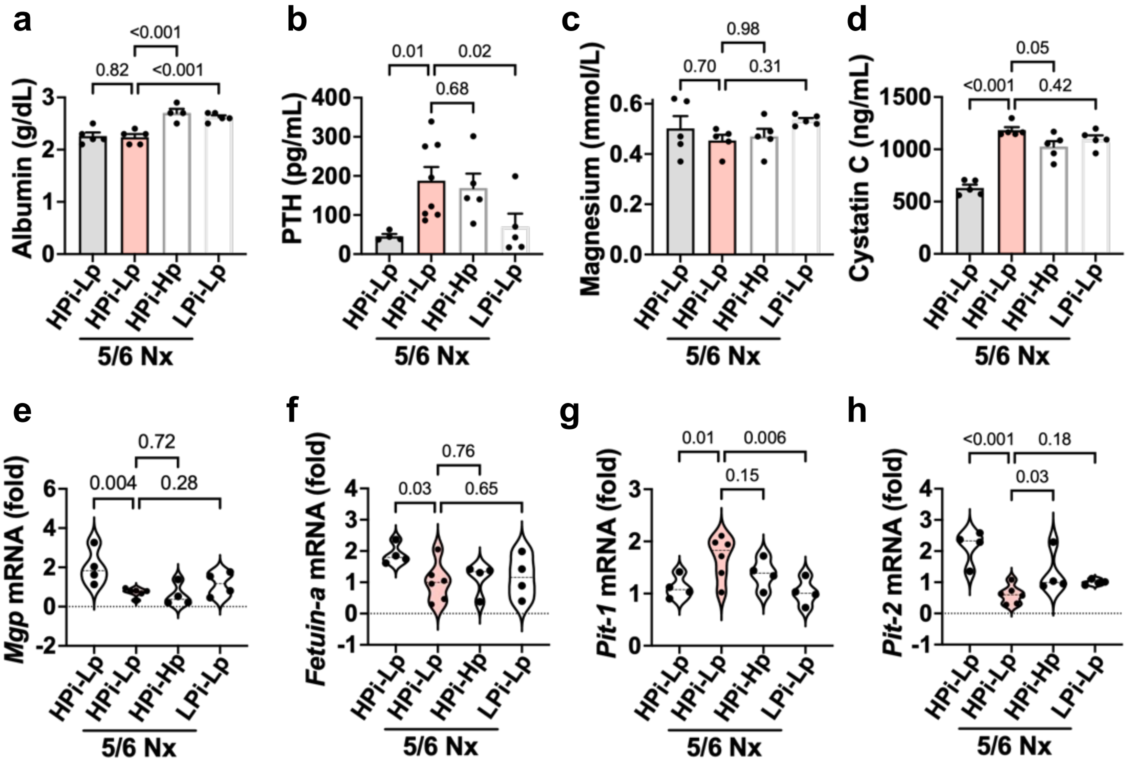

Supplemental Figure 3. Kidney dysfunction was exacerbated in the 5/6 nephrectomy mice fed the HPi-Lp diet. 12-week-old male C57BL/6J mice underwent 5/6 nephrectomy, and 2 weeks later, were fed the various diets for 28 days. (**a-d**) Biochemical analysis of albumin, PTH, magnesium and cystatin c in blood serum. *n* = 4 to 8 mice. (**e-h**) qPCR detection of *Mgp*, *Fetuin-a*, *Pit-1* and *Pit-2* in the aortas of 5/6 nephrectomy mice fed a HPi-Lp diet compared with the other groups. Gene expression levels were normalized to GAPDH and were presented as fold changes relative to the 5/6 nephrectomy-LPi-Lp group. *n* = 4 or 6. Values are mean ± SEM. Data were analyzed using one-way ANOVA adjusted with Tukey's post-hoc test for multiple comparisons. 5/6 Nx: 5/6 nephrectomy.

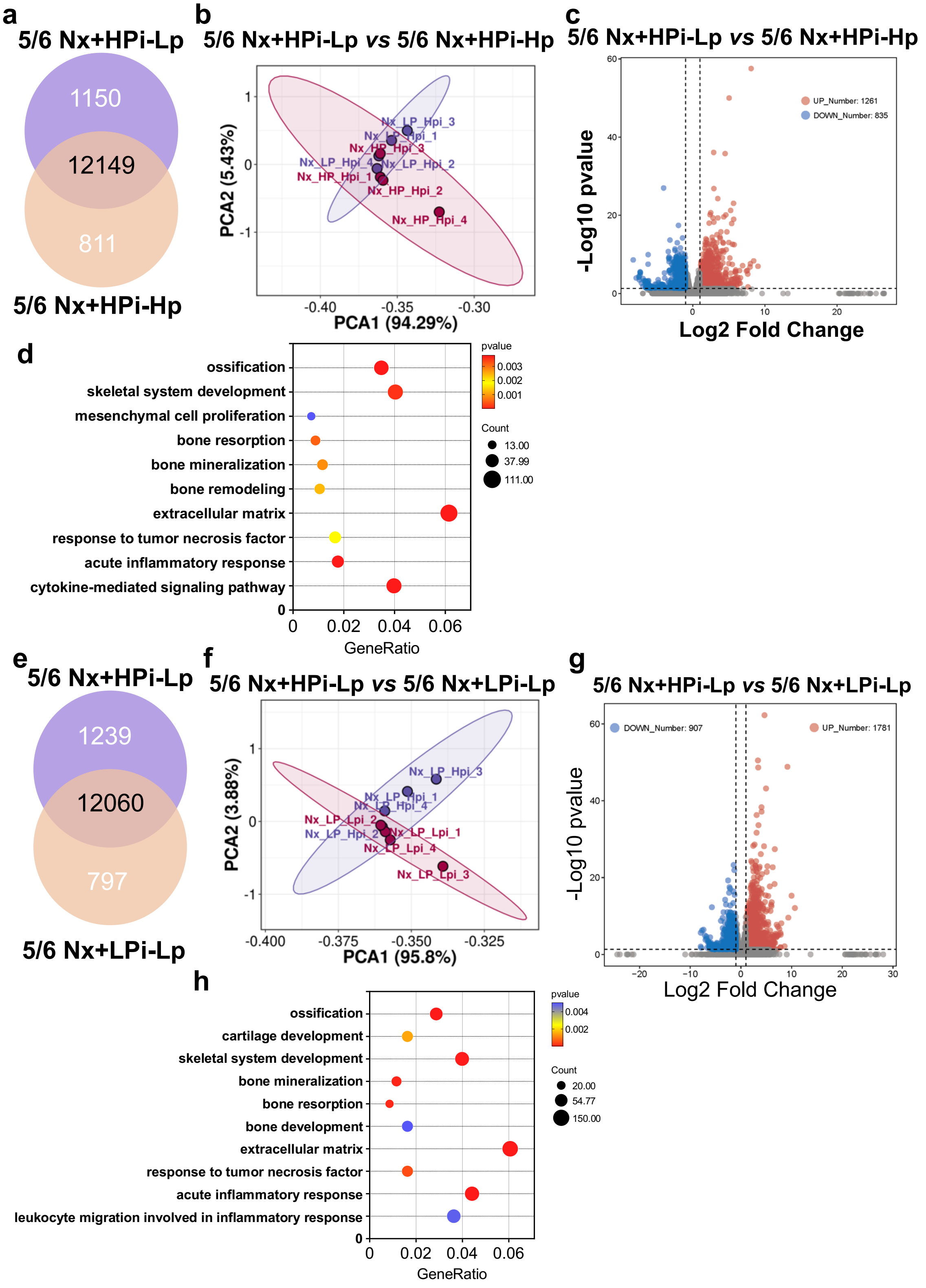
a
5/6 Nx+HPi-Lp
1150
12149
811
5/6 Nx+HPi-Hp
b
5/6 Nx+HPi-Lp vs 5/6 Nx+HPi-Hp
PCA2 (5.43%)
PCA1 (94.29%)
c
5/6 Nx+HPi-Lp vs 5/6 Nx+HPi-Hp
UP_Number: 1261
DOWN_Number: 835
-Log10 pvalue
Log2 Fold Change
d
ossification
skeletal system development
mesenchymal cell proliferation
bone resorption
bone mineralization
bone remodeling
extracellular matrix
response to tumor necrosis factor
acute inflammatory response
cytokine-mediated signaling pathway
pvalue
Count
GeneRatio
e
5/6 Nx+HPi-Lp
1239
12060
797
5/6 Nx+LPi-Lp
f
5/6 Nx+HPi-Lp vs 5/6 Nx+LPi-Lp
PCA2 (3.88%)
PCA1 (95.8%)
g
5/6 Nx+HPi-Lp vs 5/6 Nx+LPi-Lp
DOWN_Number: 907
UP_Number: 1781
-Log10 pvalue
Log2 Fold Change
h
ossification
cartilage development
skeletal system development
bone mineralization
bone resorption
bone development
extracellular matrix
response to tumor necrosis factor
acute inflammatory response
leukocyte migration involved in inflammatory response
pvalue
Count
GeneRatio

Supplemental Figure 4. RNA-seq analysis in the 5/6 nephrectomy mice fed the HPi-Lp diet, HPi-Hp diet, or LPi-Lp diet. 12-week-old male C57BL/6J mice underwent 5/6 nephrectomy, and 2 weeks later, were fed the indicated diets for 4 weeks. Total RNA was isolated from the aortas for RNA-Seq. **(a)** The Venn diagram of RNA seq data of the aortas from the 5/6 nephrectomy-HPi-Lp and the 5/6 nephrectomy-HPi-Hp mice. (**b**) PCA on the gene expression value between the 5/6 nephrectomy-HPi-Lp and the 5/6 nephrectomy-HPi-Hp mice. (**c**) The volcano plot showed 2096 significantly differentially expressed genes between the 5/6 nephrectomy-HPi-Lp and the 5/6 nephrectomy-HPi-Hp mice. (**d**) GO enrichment analysis showed multiple calcification-related pathways enriched between the 5/6 nephrectomy-HPi-Lp and the 5/6 nephrectomy-HPi-Hp mice. (**e**) The Venn diagram of RNA seq data of the aortas from the 5/6 nephrectomy-HPi-Lp and the 5/6 nephrectomy-LPi-Lp mice. (**f**) PCA on the gene expression value between the 5/6 nephrectomy-HPi-Lp and the 5/6 nephrectomy-LPi-Lp group. (**g**) Volcano plot showed 2688 significantly differentially expressed genes between the 5/6 nephrectomy-HPi-Lp and the 5/6 nephrectomy-LPi-Lp mice. (**h**) GO enrichment analysis showed multiple calcification-related pathways enriched between the 5/6 nephrectomy-HPi-Lp and the 5/6 nephrectomy-LPi-Lp group. 5/6 Nx: 5/6 nephrectomy.

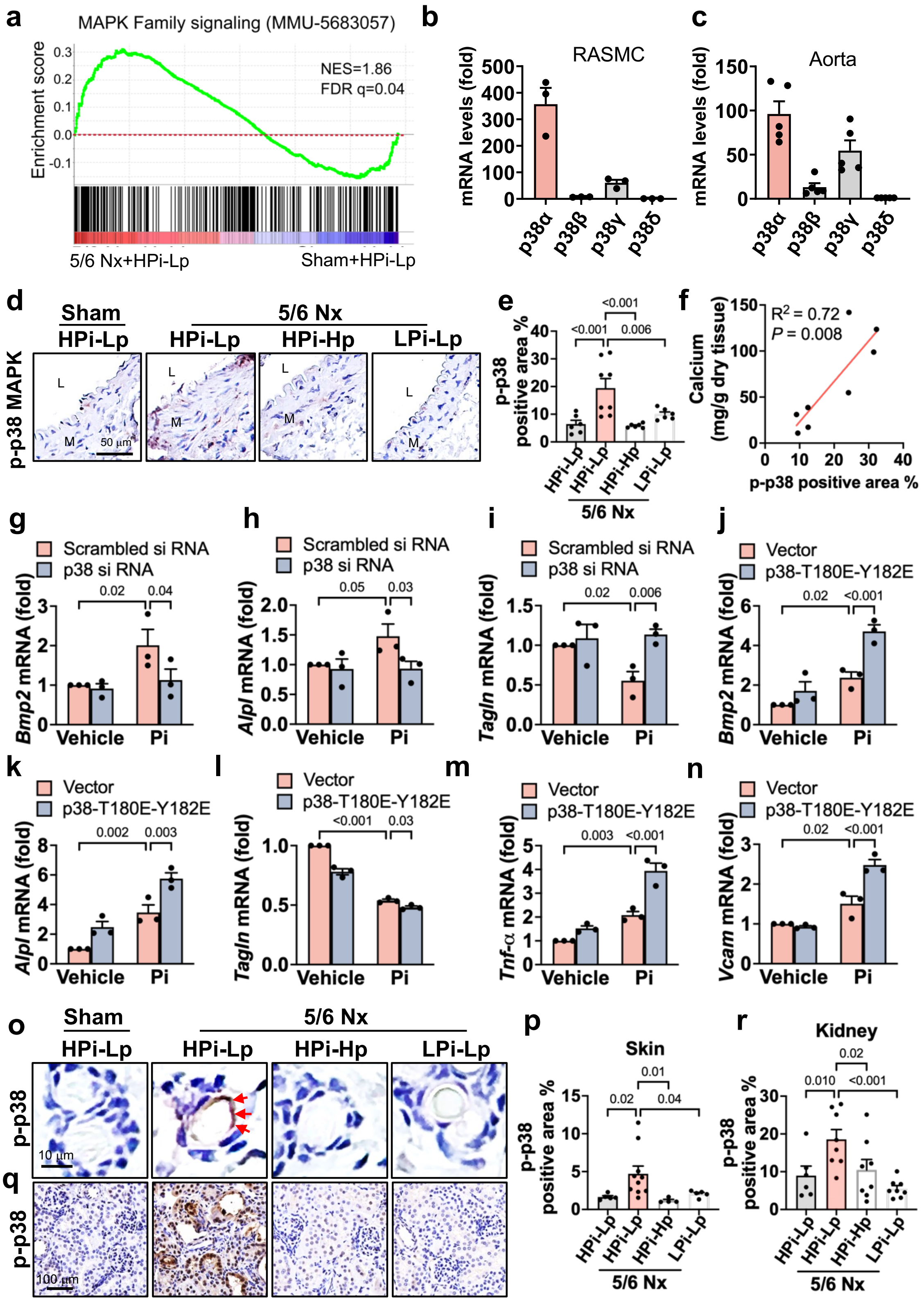
a
MAPK Family signaling (MMU-5683057)
Enrichment score
NES=1.86
FDR q=0.04
5/6 Nx+HPi-Lp
Sham+HPi-Lp
b
RASMC
mRNA levels (fold)
p38α
p38β
p38γ
p38δ
c
Aorta
mRNA levels (fold)
d
Sham
5/6 Nx
HPi-Lp
HPi-Hp
LPi-Lp
p-p38 MAPK
50 μm
e
p-p38 positive area %
<0.001
0.006
5/6 Nx
f
Calcium (mg/g dry tissue)
R^2 = 0.72
P = 0.008
p-p38 positive area %
g
Scrambled si RNA
p38 si RNA
Bmp2 mRNA (fold)
0.02
0.04
Vehicle
Pi
h
Alpl mRNA (fold)
0.05
0.03
i
Tagln mRNA (fold)
0.02
0.006
j
Vector
p38-T180E-Y182E
<0.001
k
0.002
0.003
l
m
Tnf-α mRNA (fold)
0.003
n
Vcam mRNA (fold)
o
p-p38
10 μm
q
100 μm
p
Skin
0.01
r
Kidney
0.010

Supplemental Figure 5. p38 MAPK signaling was activated in the 5/6 nephrectomy mice fed the HPi-Lp diet. (a) GSEA showed that the MAPK signaling pathway was increased in the 5/6 nephrectomy-HPi-Lp mice compared with the Sham mice. (**b and c**) MAPK14 (p-38α) was the most abundant isoform among the four p38 MAPKs in smooth muscle cells and the aortas. The isoforms of p38 MAPK were examined using qPCR. (**d and e**) Immunohistochemistry staining showed that p-p38 MAPK was markedly increased in the calcified aortas of the 5/6 nephrectomy-HPi-Lp mice. n = 6 or 8. (**f**) Correlation analysis showed a strong correlation between p-p38 MAPK and calcium deposits in the aortas. (**g-i**) The data from qPCR showed that knockdown of p38 significantly blocked Pi-mediated smooth muscle cell osteogenic transformation, showing decreased osteogenic markers *Bmp2* and *Alpl* and increased SMC markers *Tagln*. (**j-n**) The data from qPCR showed that activation of p38, by transfecting with the p38-T180E-Y182E plasmid, significantly enhanced Pi-mediated smooth muscle cell osteogenic transformation and inflammation, as evidenced by increased osteogenic markers *Bmp2* and *Alpl,* decreased SMC markers *Tagln,* and upregulation of inflammation marker *Tnf-*α and *Vcam*. (**o-r**) Immunohistochemistry staining showed that p-p38 MAPK was increased in the abdominal skin and kidney sections in the 5/6 Nephrectomy-HPi-Lp mice. n = 4 to 10 mice. Values are mean $\pm$ SEM for *in vivo* studies and mean $\pm$ SD for *in vitro* experiments. Data were analyzed using one-way or two-way ANOVA adjusted with Tukey's post-hoc test for multiple comparisons. 5/6 Nx: 5/6 nephrectomy.

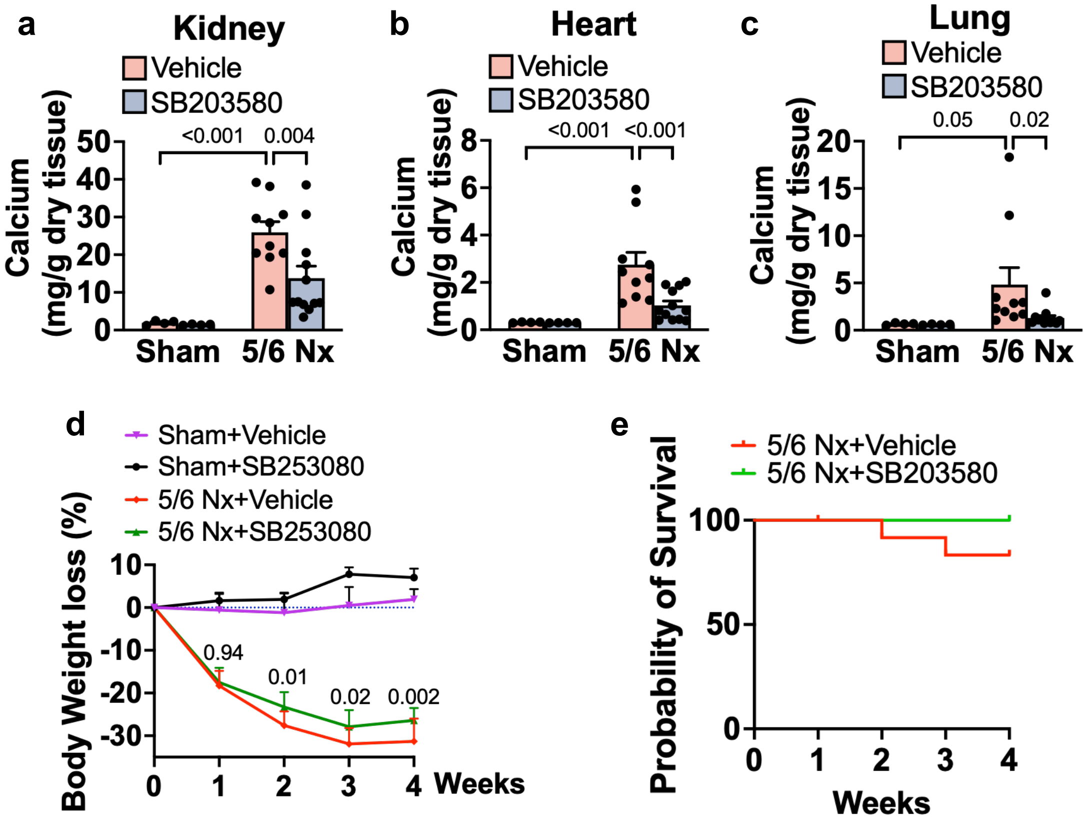

Supplemental Figure 6. Inactivation of p38 MAPK reduced organ calcification, weight loss, and animal mortality in the 5/6 nephrectomy mice fed the HPi-Lp diet. 12-week-old male C57BL/6J mice underwent 5/6 nephrectomy or Sham surgery and then IP injected with 10 mg/kg SB203580 or Vehicle, followed by a HPi-Lp diet or LPi-Hp diet feeding for 4 weeks. (**a-c**) Calcium assay showed that the administration of SB203580 significantly reduced calcium content in the kidney, heart, and lung. Sham: *n* = 4; 5/6 nephrectomy: *n* = 10-12 mice. (d) The effect of SB203580 administration on body weight in the 5/6 nephrectomy mice. (**e**) The Kaplan-Meier curve showed that the administration of SB203580 improved the survival rate of mice. n = 10 or 12 mice. Values are mean ± SEM. Data were analyzed using two-way ANOVA adjusted with Tukey's post-hoc test for multiple comparisons. 5/6 Nx: 5/6 nephrectomy.

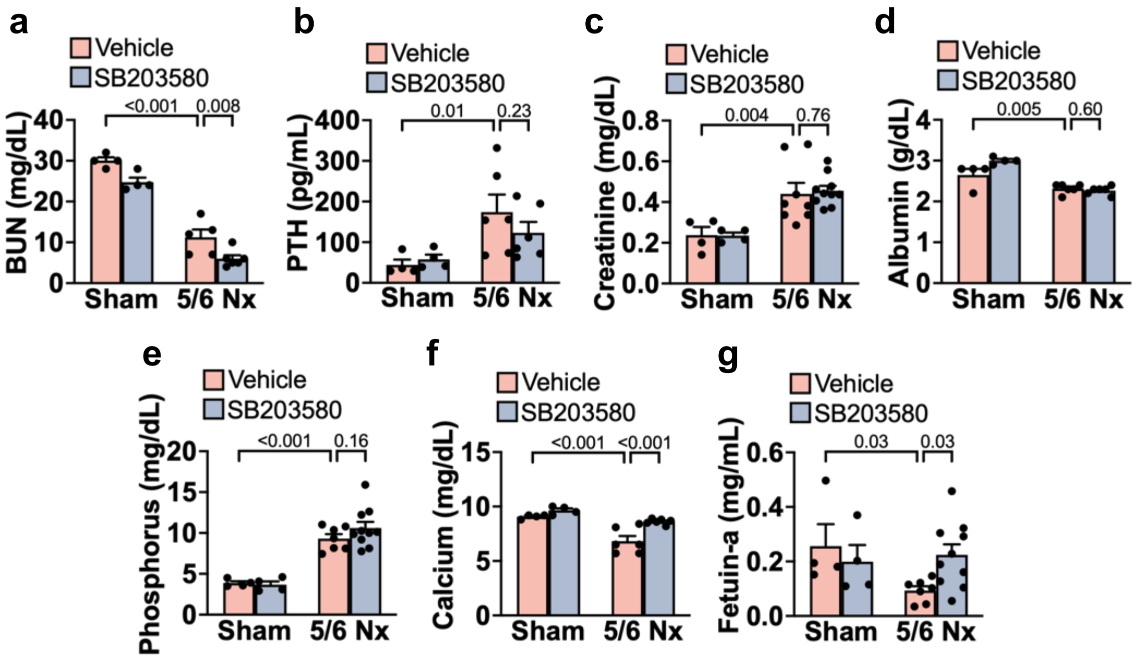

Supplemental Figure 7. Kidney function in the 5/6 nephrectomy-HPi-Lp mice after SB203580 administration. 12-week-old male C57BL/6J mice underwent 5/6 nephrectomy or Sham surgery and then IP injected with 10 mg/kg SB203580 or Vehicle, followed by a HPi-Lp diet or LPi-Hp diet feeding for 4 weeks. Blood was collected, and the kidney was isolated. (**a-g**) Biochemical analysis of BUN, PTH, creatinine, albumin, phosphorus, calcium, and fetuin-a in blood serum. *n* = 4 to 10 mice. Values are mean ± SEM. Data were analyzed using two-way ANOVA adjusted with Tukey's post-hoc test for multiple comparisons. 5/6 Nx: 5/6 nephrectomy.

References

1. Badger, A.M., *et al.* Pharmacological profile of SB 203580, a selective inhibitor of cytokine suppressive binding protein/p38 kinase, in animal models of arthritis, bone resorption, endotoxin shock and immune function. *J Pharmacol Exp Ther* **279**, 1453-1461 (1996).
2. Bain, J., *et al.* The selectivity of protein kinase inhibitors: a further update. *Biochem J* **408**, 297-315 (2007).
3. Cai, Y., *et al.* Role of cAMP-phosphodiesterase 1C signaling in regulating growth factor receptor stability, vascular smooth muscle cell growth, migration, and neointimal hyperplasia. *Circ Res* **116**, 1120-1132 (2015).
4. Cai, Y., Wang, X.-L., Flores, A.M., Lin, T. & Guzman, R.J. Inhibition of endo-lysosomal function exacerbates vascular calcification. *Scientific Reports* **8**, 3377 (2018).
5. Qin, X., Corriere, M.A., Matrisian, L.M. & Guzman, R.J. Matrix metalloproteinase inhibition attenuates aortic calcification. *Arteriosclerosis, thrombosis, and vascular biology* **26**, 1510-1516 (2006).
6. Mortazavi, A., Williams, B.A., McCue, K., Schaeffer, L. & Wold, B. Mapping and quantifying mammalian transcriptomes by RNA-Seq. *Nature methods* **5**, 621-628 (2008).
7. Liao, Y., Smyth, G.K. & Shi, W. featureCounts: an efficient general purpose program for assigning sequence reads to genomic features. *Bioinformatics* **30**, 923-930 (2014).
8. Love, M.I., Huber, W. & Anders, S. Moderated estimation of fold change and dispersion for RNA-seq data with DESeq2. *Genome biology* **15**, 1-21 (2014).
9. Kanehisa, M. & Goto, S. KEGG: kyoto encyclopedia of genes and genomes. *Nucleic acids research* **28**, 27-30 (2000).
10. McKenna, A., *et al.* The Genome Analysis Toolkit: a MapReduce framework for analyzing next-generation DNA sequencing data. *Genome research* **20**, 1297-1303 (2010).
11. Domander, R., Felder, A.A. & Doube, M. BoneJ2-refactoring established research software. *Wellcome Open Research* **6**(2021).
